# Risk of dyslipidaemia in people living with HIV who are taking tenofovir alafenamide: a systematic review and meta‐analysis

**DOI:** 10.1002/jia2.26358

**Published:** 2024-09-20

**Authors:** Jeong‐Ju Yoo, Eun Ae Jung, Sang Gyune Kim, Young Seok Kim, Min Jae Kim

**Affiliations:** ^1^ Department of Internal Medicine Soonchunhyang University Bucheon Hospital Soonchunhyang University College of Medicine Bucheon Republic of Korea; ^2^ Department of Medical Library Soonchunhyang University Bucheon Hospital Soonchunhyang University College of Medicine Bucheon Republic of Korea; ^3^ Department of Infectious Diseases Asan Medical Center University of Ulsan College of Medicine Seoul Republic of Korea

**Keywords:** cholesterol, HIV, lipid profile, meta‐analysis, tenofovir alafenamide, tenofovir disoproxil fumarate

## Abstract

**Introduction:**

Among many antiretroviral drugs, tenofovir alafenamide is used extensively in combination regimens of tenofovir/emtricitabine or tenofovir/emtricitabine/bictegravir. However, concerns have arisen about the potential of tenofovir alafenamide to exacerbate hyperlipidaemia. This meta‐analysis evaluates the relationship between tenofovir alafenamide use and lipid‐profile alterations in people living with HIV.

**Methods:**

We searched PubMed, Ovid MEDLINE, EMBASE and the Cochrane Library to identify studies on changes in cholesterol levels (e.g. total cholesterol, low‐density and high‐density lipoprotein cholesterol, and triglycerides) in people living with HIV who received treatment with a regimen containing tenofovir alafenamide (data collected 31 March 2023, review completed 30 July 2023). Potential risk factors for worsening lipid profile during treatment with tenofovir alafenamide were also evaluated.

**Results:**

Sixty‐five studies involving 39,713 people living with HIV were selected. Significant increases in total cholesterol, low‐density and high‐density lipoprotein cholesterol, and triglycerides were observed after treatment with tenofovir alafenamide. Specifically, low‐density lipoprotein cholesterol (+12.31 mg/dl) and total cholesterol (+18.86 mg/dl) increased markedly from the third month of tenofovir alafenamide use, with significant elevations observed across all time points up to 36 months. Comparatively, tenofovir alafenamide regimens resulted in higher lipid levels than tenofovir disoproxil fumarate regimens at 12 months of use. Notably, discontinuation of the tenofovir alafenamide regimen led to significant decreases in low‐density lipoprotein cholesterol (–9.31 mg/dl) and total cholesterol (–8.91 mg/dl). Additionally, tenofovir alafenamide use was associated with increased bodyweight (+1.38 kg; 95% confidence interval: 0.92–1.84), which became more pronounced over time. Meta‐regression analysis identified young age, male sex and low body mass index as risk factors for worsening cholesterol levels in individuals treated with tenofovir alafenamide.

**Conclusions:**

Tenofovir alafenamide use in people living with HIV is associated with significant alterations in lipid profile.

## 1 INTRODUCTION

The management of human immunodeficiency virus (HIV) infection has witnessed transformative advancements since the advent of antiretroviral therapy (ART), revolutionizing the prognosis for individuals diagnosed with HIV. The development and optimization of antiretroviral regimens have not only prolonged life expectancy but have also significantly enhanced the quality of life for people living with HIV, turning what was once considered a fatal diagnosis into a manageable chronic condition. Currently, combination drugs are widely used due to their convenience and improved drug adherence. Among these drugs, tenofovir takes particular importance since it is not only considered for ordinary HIV treatment but also for person living with HIV and hepatitis B virus and for individuals requiring pre‐exposure prophylaxis [1, 2, 3]. The adoption of tenofovir alafenamide (TAF) into antiretroviral regimens was largely driven by reduced nephrotoxicity and a lower impact on bone density than the forerunner tenofovir disoproxil fumarate (TDF) [4, 5]. Despite these advantages of TAF, emerging concerns regarding the potential impact of TAF on metabolic health, particularly lipid metabolism, have garnered attention within the medical and research communities, prompting a need for comprehensive evaluation [6, 7].

In chronic hepatitis B, another condition in which TAF is used, a meta‐analysis demonstrated that dyslipidaemia worsened following the use of TAF [8, 9]. Dyslipidaemia, manifesting as increased levels of lipids such as cholesterol and triglycerides in the bloodstream, is a significant risk factor for cardiovascular disease (CVD), a leading cause of morbidity and mortality worldwide [10]. In the context of HIV treatment, the long‐term use of ART has been associated with metabolic disturbances, including dyslipidaemia, which complicates the management of people living with or persons living with HIV by increasing the risk of CVD [11]. The incorporation of TAF into treatment protocols has been linked to altered lipid profiles, raising critical questions about the metabolic safety and potential role of TAF in exacerbating dyslipidaemia in people living with or persons living with HIV. Therefore, the aim of this meta‐analysis was to determine the extent to which TAF is associated with worsening dyslipidaemia in individuals undergoing HIV treatment, by synthesizing existing research on the impact of TAF on lipid levels.

## 2 METHODS

This systematic review and meta‐analysis adhered to the Preferred Reporting Items for Systematic Reviews and Meta‐Analyses (PRISMA) guidelines and the Meta‐analysis of Observational Studies in Epidemiology (MOOSE) checklist, and was registered beforehand with PROSPERO (International Prospective Register of Systematic Reviews, CRD42024513906). Ethics approval was waived from the Institutional Review Board and our study conformed to the ethical guidelines of the World Medical Association Declaration of Helsinki. Informed consent was waived due to meta‐analysis.

### 2.1 Inclusion criteria, exclusion criteria and study outcomes

We included randomized controlled trials (RCTs), and prospective and retrospective cross‐sectional or cohort studies that examined changes in lipid profiles in adults (aged ≥18 years) undergoing treatment with regimens containing TAF. Our inclusion criteria did not limit studies based on the duration of treatment, ethnicity or geographical location. For studies where only a subset of participants were switched from TAF to a non‐TAF regimen, we included the study if we could extract information specifically for the subgroup that used TAF. If at least one lipid profile parameter was reported with exact numerical values, the study was included. We excluded case reports, case series with fewer than five patients, review articles, studies involving people living with or persons living with HIV and hepatitis B virus or hepatitis C virus, and studies that did not report specific lipid‐profile values or presented values only as ratios. If all lipid profiles were not reported, the study was excluded. We focused on individuals with HIV who are on ART. Studies involving the prophylaxis group were excluded from our analysis. The primary objective of the study was to assess the extent of lipid‐profile alterations following treatment with TAF. This assessment considered lipid‐profile changes in three clinical scenarios: (1) before and after TAF treatment in the same individuals; (2) comparing TAF‐based regimens with TDF‐based regimens; and (3) following the discontinuation of TAF. Subgroup analyses were conducted based on various factors, such as study design, income level, age, sex distribution, year of publication, sample size and the presence of commercial funding. Additionally, the secondary objective was to identify risk factors for changes in total cholesterol or low‐density lipoprotein (LDL)‐cholesterol due to TAF use. This involved compiling all factors associated with cholesterol changes from each study and conducting a meta‐regression analysis on the factors frequently mentioned across multiple studies.

### 2.2 Search strategy

The search strategy encompassed terms related to HIV, cholesterol and TAF. Synonyms for these terms were identified and used to formulate the search approach. The specific keywords employed within the framework of the Patient/Problem, Intervention, Comparison, and Outcome (PICO) model are detailed in the Supplementary Materials. We conducted searches in databases including Medline (PubMed), EMBASE, Cochrane Library, Web of Science and KoreaMed, using Medical Subject Headings (MeSH) to find studies published in English from 1 January 2005 to 28 February 2023. Our data were collected on 31 March 2023, and the data review was completed on 30 July 2023. The methods section and Supplementary Materials provide details on the search strategies and the outcomes from each database search. A professional librarian (EAJ) executed all search activities.

### 2.3 Study selection and data extraction

Titles and abstracts were independently reviewed by two authors, while full‐text articles were independently assessed for their relevance to the study by two reviewers (J‐JY and MJK). In cases of disagreement between the reviewers, SKK resolved the discrepancies through discussion. Additionally, both researchers independently conducted a risk of bias evaluation for all studies included, and also extracted and documented the characteristics and findings of these studies using a standardized format.

### 2.4 Methodological quality and risk of bias assessment

The evaluation of bias risk varied based on the type of each study. For randomized trials, the assessment was conducted using the Cochrane risk of bias tool. Meanwhile, for non‐randomized studies, such as cohort studies, the Risk of Bias Assessment tool for Non‐randomized Studies (RoBANS) was employed. The detailed outcomes of these assessments are available in the risk of bias section in the Supplementary Materials. Disagreements between the two authors (J‐JY and MJK) were settled through discussion. To evaluate publication bias, funnel plots were used.

### 2.5 Statistical analyses

Analyses of lipid‐profile changes before and after treatment with TAF, and comparisons between the TAF‐regimen and TDF‐regimen groups, were presented as mean differences for continuous variables and as Freeman‐Tukey variants for binary variables. Estimations of variance between studies were performed using the DerSimonian‐Laird method. To assess heterogeneity across studies, we used the *I* ^2^ metric, which quantifies the proportion of total variation across studies due to heterogeneity rather than chance, and the *p*‐value from Cochran's *Q* test. The *I* ^2^ values can range from 0% (no observed heterogeneity) to 100% (maximum heterogeneity). Publication bias was assessed using the AS‐Thompson test. For the statistical analyses, we used RevMan 5 software from the Cochrane Library and the meta package in R software (version 4.1.0; R Foundation for Statistical Computing, Vienna, Austria).

Subgroup analyses were conducted to assess the impact of TAF on dyslipidaemia across various groups. Relevant definitions for subgroup analyses included high‐risk versus low‐risk ART regimens, treatment‐experienced versus treatment‐naïve, age groups, sex distribution, income level of study country, funding source and sample size. High‐risk regimens included those containing protease inhibitors or elvitegravir, which are known to significantly affect lipid profiles. Low‐risk regimens did not include these agents.

To identify risk factors associated with worsening lipid profiles in patients taking TAF, we performed meta‐regression analyses using study‐level factors. These factors included the average age of participants, average body mass index (BMI) of participants, the percentage of study participants with prior dyslipidaemia and other relevant study‐level characteristics. Each covariate was included in the meta‐regression model to adjust for its potential confounding effect on the lipid outcomes. We used the DerSimonian‐Laird method for random‐effects meta‐regression to account for between‐study heterogeneity. The number of models fit corresponded to the number of covariates evaluated, with each model assessing the independent effect of one covariate on the lipid outcomes. We reported the regression coefficients, 95% confidence intervals (CIs) and *p*‐values for each covariate to determine its significance. Additionally, we evaluated the proportion of variance explained (*R* ^2^) by each model to understand the contribution of each covariate to the observed lipid changes.

## 3 RESULTS

### 3.1 Characteristics of included studies

**Figure 1:**
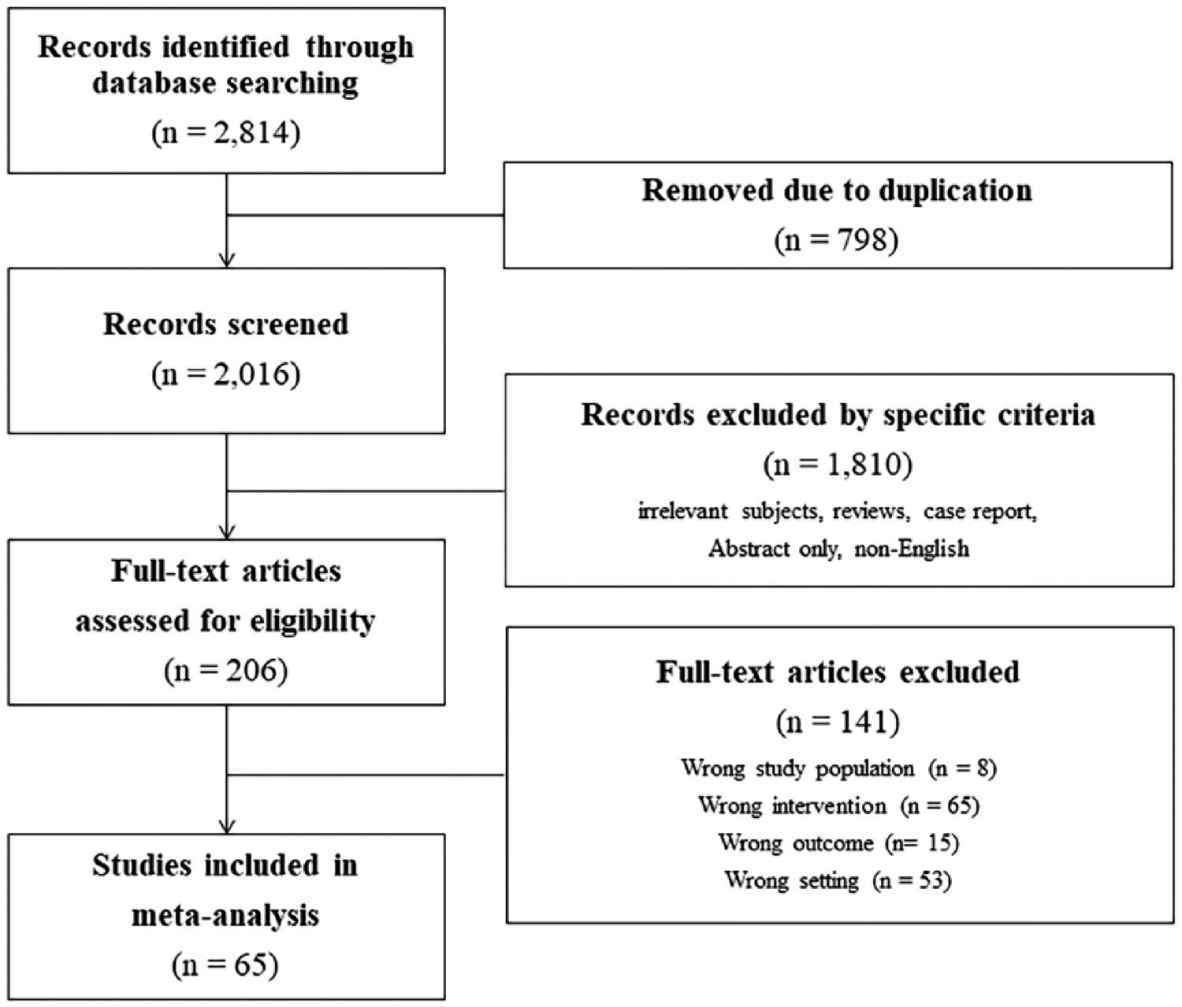
Flow charts of the study.

Ultimately, 65 studies were included for analysis (Figure 1). Information on the enrolled patients is presented in Table 1. Among the studies included, 35 were prospective, while 30 were retrospective. Twenty‐one studies were RCTs, and 44 were non‐RCTs, including cohort studies. Sixteen studies focused on treatment‐naive patients, 47 targeted treatment‐experienced patients, and the remaining two studies included both treatment‐naive and treatment‐experienced patients. The average age of study participants ranged from 31 to 69 years, with 63 studies predominantly involving male participants. The included studies were conducted across various geographical regions, reflecting a diverse population of people living with or persons living with HIV. The majority of the studies were from high‐income countries, particularly the United States and countries in Europe, while a smaller number of studies were conducted in Asia, Africa and South America.

**Table 1:** Characteristics of the included studies

Author	Year	Study design	Study design	Hospitals	Country	Number of patients	Treatment experience	Age (years)	Male (%)
Abe et al. [12]	2021	Retrospective	Cohort	Single	Japan	70	Experienced	44	92.9
Adachi et al. [13]	2022	Retrospective	Cohort	Single	Japan	119	Naïve	50	97
Alejos et al. [14]	2020	Retrospective	Cohort	Multi	Spain	446	Naïve	36	88.5
Atencio et al. [15]	2021	Prospective	Cohort	Multi	Spain	22	Naïve	34.8	100
Baldin et al. [16]	2021	Retrospective	Cohort	Single	Italy	126	Experienced	53.1	71.4
Bendala‐Estrada et al. [17]	2022	Retrospective	Cohort	Single	Spain	118	Naïve	51	86.4
Bosch et al. [18]	2023	Prospective	RCT	Multi	South Africa	70	Naïve	33	41
Brunet et al. [19]	2021	Prospective	Cohort	Multi	USA	6451	Experienced	47.9	84.3
Cid‐Silva et al. [20]	2019	Retrospective	Cohort	Single	Spain	233	Naïve and experienced	46.5	75.5
Daar et al. [21]	2018	Prospective	RCT	Multi	Multi	290	Experienced	48	84
Dejesus et al. [22]	2018	Prospective	RCT	Multi	USA	959	Experienced	41	89.3
Eron et al. [23]	2019	Prospective	RCT	Multi	USA and Europe	1141	Experienced	46	82
Eron et al. [24]	2018	Prospective	RCT	Multi	USA and Europe	362	Naïve	34	88
Funderburg et al. [25]	2016	Prospective	RCT	Multi	USA	98	Naïve	34	89
Gallant et al. [26]	2017	Prospective	RCT	Multi	USA and Europe	314	Naïve	31	91
Gazzola et al. [7]	2021	Retrospective	Cohort	Single	Italy	221	Experienced	45	78.7
Giacomelli et al. [27]	2021	Retrospective	Cohort	Single	Italy	261	Experienced	48	75.9
Gilbert et al. [28]	2022	Retrospective	Cohort	Single	USA	142	Experienced	66	90
Hagins et al. [29]	2021	Prospective	RCT	Multi	USA	495	Experienced	49	69
Huhn et al. [30]	2019	Prospective	RCT	Multi	USA, Europe and Asia	866	Naïve	33	85
Ikeda et al. [31]	2021	Prospective	Cohort	Single	Japan	31	Experienced	45	100
Kanda et al. [32]	2021	Prospective	Cohort	Single	Japan	118	Experienced	44	94
Kauppinen et al. [33]	2022	Retrospective	Cohort	Single	Finland	146	Naïve	50	79
Kauppinen et al. [34]	2019	Retrospective	Cohort	Single	Finland	490	Experienced	48	76
Kim et al. [35]	2022	Retrospective	Cohort	Multi	Korea	191	Experienced	43	90.6
Kityo et al. [36]	2019	Prospective	RCT	Multi	USA, Europe and Asia	234	Experienced	39	0
Kovari et al. [37]	2021	Prospective	Cohort	Multi	Switzerland	1712	Experienced	50	75
Kuo et al. [38]	2020	Retrospective	Cohort	Single	Taiwan	693	Experienced	43.9	95.1
Lacey et al. [39]	2020	Retrospective	Cohort	Single	Ireland	194	Experienced	46	70.6
Lagoutte‐Renosi et al. [40]	2021	Retrospective	Cohort	Single	France	103	Experienced	51	72.8
Lazzaro et al. [41]	2022	Retrospective	Cohort	Single	Italy	147	Experienced	57	70.7
Maggiolo et al. [42]	2021	Prospective	Single arm	Multi	USA, Europe and Asia	86	Experienced	69	87.2
Maggiolo et al. [43]	2019	Prospective	RCT	Multi	USA, Europe and Asia	110	Experienced	65	87
Mallon et al. [6]	2021	Prospective	Cohort	Multi	USA	6451	Experienced	48	84
Martínez‐Sanz et al. [44]	2023	Prospective	Cohort	Multi	Spain	1955	Experienced	38	86
Mazzitelli et al. [45]	2022	Prospective	Cohort	Single	Italy	290	Experienced	52	76.9
Milinkovic et al. [46]	2019	Retrospective	Cohort	Single	UK	385	Experienced	49	90
Molina et al. [47]	2018	Prospective	RCT	Multi	USA and Europe	282	Experienced	47	88
Moschopoulos et al. [48]	2023	Retrospective	Cohort	Single	Greece	62	Experienced	32.9	98.4
Orkin et al. [49]	2020	Prospective	RCT	Multi	USA and Europe	959	Naïve	31	91
Orkin et al. [50]	2020	Prospective	RCT	Multi	USA and Europe	725	Naïve and experienced	34	88
Orkin et al. [51]	2018	Prospective	RCT	Multi	USA and Europe	763	Experienced	46	82
Petrakis et al. [52]	2020	Retrospective	Cohort	Single	Greece	85	Experienced	43.91	82.9
Plum et al. [53]	2021	Retrospective	Cohort	Single	Belgium	98	Experienced	50	64.3
Podzamczer et al. [54]	2021	Prospective	RCT	Multi	Spain	151	Naïve	34	97
Rizzardini et al. [55]	2019	Prospective	RCT	Multi	USA and Europe	183	Experienced	49	85
Rolle et al. [56]	2020	Retrospective	Cohort	Single	USA	61	Experienced	53	77
Rolle et al. [57]	2021	Retrospective	Cohort	Single	USA	350	Experienced	57	80
Sax et al. [58]	2014	Prospective	RCT	Multi	USA	112	Naïve	34	96
Schafer et al. [59]	2019	Retrospective	Cohort	Single	USA	110	Experienced	50	72.7
Schafer et al. [60]	2022	Retrospective	Cohort	Single	USA	86	Experienced	47.1	67.4
Schwarez‐Zander et al. [61]	2020	Retrospective	Cohort	Single	Germany	347	Experienced	49	73
Sekiya et al. [62]	2023	Retrospective	Cohort	Single	Japan	328	Experienced	40.9	98.4
Shokoohi et al. [63]	2021	Retrospective	Cohort	Single	Canada	651	Experienced	48.2	92.9
Squillace et al. [64]	2020	Prospective	Cohort	Multi	Italy	190	Experienced	46.7	80
Sun et al. [65]	2022	Retrospective	Cohort	Single	China	91	Naïve	32	91.2
Surial et al. [66]	2021	Prospective	Cohort	Multi	Switzerland	3484	Experienced	50	75.3
Tabak et al. [67]	2020	Prospective	Cohort	Multi	Turkey	614	Experienced	42	89
Taramasso et al. [68]	2019	Retrospective	Cohort	Multi	Italy	573	Experienced	49.7	74.7
Van Wyk et al. [69]	2020	Prospective	RCT	Multi	USA and Europe	372	Naïve	40	93.2
Verburgh et al. [70]	2022	Prospective	Cohort	Multi	Netherland	1544	Experienced	51.5	85.6
Walti et al. [71]	2018	Retrospective	Cohort	Single	Switzerland	10	Experienced	55	90
Winston et al. [72]	2018	Prospective	RCT	Multi	USA and Europe	280	Experienced	52	86
Wohl et al. [73]	2016	Prospective	RCT	Multi	USA, Europe and Asia	866	Naïve	33	85
Xia et al. [74]	2021	Prospective	Cohort	Single	China	196	Experienced	37.5	96.9

Abbreviation: RCT, randomized controlled trial.

The heterogeneity among the included studies was assessed using the *I* ^2^ statistic. The *I* ^2^ values ranged from moderate to high for various lipid parameters, indicating substantial heterogeneity. Specifically, the *I* ^2^ values were 86% for high‐density lipoprotein (HDL)‐cholesterol at 3 months, 93% for LDL‐cholesterol at 3 months and 96% for total cholesterol at 3 months, suggesting considerable variability among the studies. Regarding the risk of bias, most RCTs were assessed as having a low risk of bias, while the cohort studies varied, with some showing a higher risk of bias due to potential confounding factors and selection bias (Supplementary Materials). For publication bias, the funnel plots for HDL‐cholesterol, LDL‐cholesterol, total cholesterol and triglycerides did not show significant asymmetry, suggesting a low likelihood of publication bias. The AS‐Thompson test results further supported this conclusion, indicating no significant evidence of publication bias in the included studies (Figures S1–S4).

### 3.2 Changes in lipid profile before and after using a TAF‐regimen

Initially, we analysed the lipid‐profile changes before and after implementation of a TAF‐based regimen (Table 2). Compared to baseline levels before TAF administration, HDL‐cholesterol increased significantly at all observed time points after starting TAF. Three months post‐TAF initiation, the increase in HDL‐cholesterol (ΔHDL) was 3.89 mg/dl (95% CI: 2.50–5.27), and at 12 months, ΔHDL was 2.47 mg/dl (95% CI: 1.66–3.29). For LDL cholesterol, there was a notable increase starting from the third month of TAF treatment, with a mean difference of 12.31 mg/dl, and at all subsequent time points up to 36 months, LDL cholesterol levels were significantly higher than baseline. Similarly, total cholesterol levels began to increase, with a mean difference of 18.86 mg/dl (95% CI: 10.49–27.23) from the third month of TAF treatment. Total cholesterol levels remained significantly elevated compared to baseline at all time points: 6, 12 and 24 months. Like the other lipid parameters, triglycerides also increased significantly after TAF administration compared to baseline levels. The study names and forest plots used in each analysis are presented in Figure S1.

**Table 2:** Lipid‐profile changes during TAF treatment (vs. baseline)

Outcome	Time point	No. of studies	Mean difference	95% CI	*I* ^2^	*p* for heterogeneity
HDL‐cholesterol	3 months	9	3.89	2.50–5.27	86	<0.001
	6 months	18	3.54	2.19–4.89	94	<0.001
	12 months	36	2.47	1.66–3.29	92	<0.001
	24 months	13	3.40	2.95–3.84	54	0.009
	36 months	5	3.17	1.64–4.70	87	<0.001
LDL‐cholesterol	3 months	11	12.31	7.78–16.84	93	<0.001
	6 months	22	10.27	6.66–13.89	93	<0.001
	12 months	39	9.06	6.39–11.72	95	<0.001
	24 months	13	13.00	7.67–18.33	96	<0.001
	36 months	5	13.03	5.77–20.30	96	<0.001
Total cholesterol	3 months	11	18.86	10.49–27.23	96	<0.001
	6 months	20	18.86	12.54–25.17	94	<0.001
	12 months	40	12.90	8.89–16.90	97	<0.001
	24 months	14	19.47	14.93–24.02	95	<0.001
	36 months	5	9.46	4.62–14.29	86	<0.001
Triglycerides	3 months	10	17.16	12.14–22.17	75	<0.001
	6 months	20	14.04	8.28–19.79	85	<0.001
	12 months	37	9.69	5.48–13.89	92	<0.001
	24 months	14	19.55	12.87–26.23	92	<0.001
	36 months	6	3.32	–0.73 to 7.37	47	0.092
Total cholesterol/HDL‐cholesterol ratio	3 months	3	0.02	–0.17 to 0.21	97	<0.001
	6 months	7	0.07	0.01–0.12	73	0.001
	12 months	30	0.06	0.01–0.12	100	<0.001
	24 months	7	0.22	0.11–0.34	91	<0.001
	36 months	4	–0.04	–0.10 to 0.02	76	0.005

Abbreviations: CI, confidence interval; HDL, high‐density lipoprotein; LDL, low‐density lipoprotein; TAF, tenofovir alafenamide.

### 3.3 Subgroup analyses in various situations, including ART risk group

We conducted analyses to assess the impact of TAF on dyslipidaemia across various subgroups (Table 3). While varying in degree, total cholesterol, LDL‐cholesterol and triglycerides increased significantly in all subgroups after 1 year of TAF use compared to baseline. Additionally, subgroup analyses were performed to examine the influence of other regimens that could affect dyslipidaemia, alongside TAF (Table 4). Regimens classified as high‐risk for causing dyslipidaemia included protease inhibitors or elvitegravir. Exacerbations of dyslipidaemia were noted both for high‐risk and low‐risk regimens, affecting both treatment‐naive and treatment‐experienced groups equally.

**Table 3:** Pooled estimates and meta‐regression analyses (by different subgroups) of lipid‐profile changes after 1 year of TAF treatment

	Total cholesterol				LDL‐cholesterol				Triglycerides				HDL‐cholesterol				Total cholesterol/HDL‐cholesterol ratio			
	*n*	Mean difference (95% CI)	Meta‐regression		*n*	Mean difference (95% CI)	Meta‐regression		*n*	Mean difference (95% CI)	Meta‐regression		*n*	Mean difference (95% CI)	Meta‐regression		*n*	Mean difference (95% CI)	Meta‐regression	
			*p*	*R* ^2^ (%)			*p*	*R* ^2^ (%)			*p*	*R* ^2^ (%)			*p*	*R* ^2^ (%)			*p*	*R* ^2^ (%)
Overall	40	12.90 (8.89–16.90)			39	9.06 (6.37–11.72)			37	9.69 (5.48–13.89)			36	2.47 (1.66–3.29)			30	0.06 (0.01–0.12)		
Study design																				
Prospective	25	14.28 (9.27–19.29)			25	9.52 (6.36–12.69)			22	9.65 (4.72–14.58)			22	2.44 (1.37–3.51)			19	0.07 (0.00–0.14)		
Retrospective	15	9.91 (3.84–15.97)	0.357	3.0	14	8.27 (3.27–13.27)	0.569	3.0	15	10.66 (1.19–20.12)	0.998	0	14	2.31 (1.33–3.28)	0.799	0	11	0.01 (–0.02 to 0.04)	0.564	40.3
Study design																				
RCT	19	14.99 (9.05–20.94)			18	9.51 (5.61–13.42)			14	8.80 (2.66–14.95)			17	2.39 (1.15–3.63)			17	0.19 (0.18–0.19)		
Non‐RCT	21	10.10 (5.24–14.94)	0.319	2.6	21	8.42 (4.81–12.04)	0.777	0	23	11.24 (4.50–17.98)	0.661	0	19	2.43 (1.44–3.41)	0.740	0	13	0.00 (0.00–0.00)	0.188	41.1
Income group																				
High	36	11.88 (7.61–16.14)			35	8.27 (5.39–11.15)			33	8.56 (3.83–13.29)			32	2.19 (1.33–3.04)			28	0.06 (0.00–0.11)		
Low/middle	4	28.33 (6.52–50.14)	0.357	3.09	4	22.48 (6.81–38.15)	0.027	0	4	24.51 (0.72–48.30)	0.083	0	4	5.72 (2.50–8.94)	0.026	0	2	0.07 (–0.11 to 0.27)	0.882	0
Age group																				
< 40 years	18	18.79 (12.71–24.86)			16	11.79 (7.96–15.61)			14	6.68 (5.36–8.00)			14	3.66 (2.52–4.80)			14	0.05 (0.01–0.08)		
≥40 years	22	8.00 (3.03–12.97)	0.008	7.5	13	7.29 (3.48–11.09)	0.074	0	23	1.95 (0.34–3.56)	0.104	0	22	1.63 (0.64–2.62)	0.007	16.6	16	0.05 (–0.02 to 0.13)	0.809	35.3
Males																				
≥80%	30	15.20 (10.65–19.76)			30	10.70 (7.91–13.49)			28	12.64 (7.95–17.34)			26	2.89 (1.96–3.82)			23	0.08 (0.01–0.14)		
<80%	10	3.54 (–1.25 to 8.33)	0.026	21.7	9	1.42 (–2.09 to 4.94)	0.004	27.0	9	–1.37 (–8.02 to 5.27)	0.006	12.5	10	1.14 (–0.18 to 2.46)	0.067	11.2	7	0.00 (–0.06 to 0.07)	0.206	0
Years published																				
2014–2019	17	16.48 (10.10–22.86)			17	10.88 (6.62–15.14)			14	9.76 (2.81–16.71)			16	3.01 (1.65–4.37)			16	0.07 (0.00–0.15)		
2020–2023	23	10.20 (5.15–15.25)	0.137	3.7	22	7.69 (4.23–11.14)	0.275	0	23	9.75 (4.13–15.36)	0.954	0	20	2.05 (1.05–3.06)	0.345	0	14	0.04 (0.01–0.08)	0.640	34.2
PI‐based ART																				
No	29	13.69 (8.51–18.88)			25	9.03 (5.56–12.49)			25	4.56 (3.27–5.84)			26	2.81 (1.79–3.83)			19	0.03 (0.00–0.06)		
Yes	11	11.66 (4.36–18.96)	0.704	0	14	9.29 (4.76–13.82)	0.945	0	13	5.14 (3.46–6.82)	0.870	0	10	1.77 (0.29–3.26)	0.276	0	11	0.11 (0.02–0.20)	0.130	44.5
Sample size																				
<200	20	14.71 (9.01–20.40)			18	12.34 (7.51–17.17)			18	14.02 (5.29–22.75)			17	2.78 (1.77–3.80)			14	0.01 (–0.01 to 0.04)		
200–500	14	7.74 (1.16–14.31)			14	5.21 (1.16–9.26)			14	4.82 (–1.85 to 11.50)			6	3.16 (2.22–4.11)			5	0.18 (0.15–0.22)		
≥500	6	20.05 (18.14–21.97)	0.279	0	7	11.59 (6.83–16.35)	0.967	0	5	15.01 (6.24–23.79)	0.695	0	13	1.50 (0.03–2.96)	0.530	0	11	0.06 (–0.02 to 0.15)	0.259	89.8
Funding																				
Non‐SIT	16	12.95 (8.55–17.35)			15	9.75 (6.32–13.18)			17	12.74 (5.81–19.67)			13	2.44 (1.55–3.33)			12	0.01 (0.00–0.02)		
SIT	24	12.23 (6.46–17.99)	0.540	0	24	8.24 (4.62–11.86)	0.339	0	20	7.94 (2.19–13.68)	0.273	0	23	2.37 (1.27–3.48)	0.578	0	18	0.05 (–0.02 to 0.14)	0.638	81.6
Treatment‐experience																				
Experienced	24	8.39 (3.21–13.57)			25	7.62 (4.19–11.05)			25	5.90 (1.24–10.55)			24	1.49 (0.60–2.38)			18	0.06 (0.00–0.14)		
Naïve	16	20.74 (13.84–27.64)	0.005	0	14	12.12 (7.53–16.72)	0.118	0	12	15.76 (9.57–21.95)	0.013	26.0	12	4.49 (3.32–5.66)	<0.001	24.3	12	0.03 (0.00–0.07)	0.774	1.7

Abbreviations: ART, antiretroviral therapy; CI, confidence interval; LDL, low‐density lipoprotein; PI, protease inhibitor; RCT, randomized controlled trial; SIT, sponsor‐initiated trial; TAF, tenofovir alafenamide.

**Table 4:** Pooled estimates of lipid‐profile changes (by different drug regimen) after 1 year of TAF treatment

	Total cholesterol				LDL‐cholesterol				Triglycerides				HDL‐cholesterol				Total cholesterol/HDL‐cholesterol ratio			
	*n*	Mean difference (95% CI)	*I* ^2^	*p* for heterogeneity	*n*	Mean difference (95% CI)	*I* ^2^	*p* for heterogeneity	*n*	Mean difference (95% CI)	*I* ^2^	*p* for heterogeneity	*n*	Mean difference (95% CI)	*I* ^2^	*p* for heterogeneity	*n*	Mean difference (95% CI)	*I* ^2^	*p* for heterogeneity
**Treatment naïve**																				
High‐risk regimen	14	19.82 (12.37–27.26)	98	<0.001	13	12.97 (7.63–18.32)	95	<0.001	10	15.49 (8.34–22.64)	87	<0.001	11	4.46 (3.15–5.77)	75	<0.001	11	0.05 (0.01–0.09)	88	<0.001
Low‐risk regimen	2	15.54 (9.40–21.68)	70	0.073	NA	NA	NA	NA	2	19.84 (–5.12 to 44.80)	82	0.028	1	5.00 (3.94–6.06)	NA	NA	1	–0.10 (–0.17 to –0.03)	NA	NA
**Treatment‐experienced**																				
Previous PI (–), Current PI (–)	6	16.43 (7.08–25.77)	96	<0.001	6	13.24 (7.21–19.27)	93	<0.001	6	10.22 (1.66–18.78)	89	<0.001	6	2.58 (0.78–4.38)	91	<0.001	5	0.08 (–0.01 to 0.17)	91	<0.001
Previous PI (–), Current PI (+)	NA	NA	NA	NA	NA	NA	NA	NA	NA	NA	NA	NA	NA	NA	NA	NA	NA	NA	NA	NA
Previous PI (+), Current PI (–)	8	2.24 (–3.95 to 8.44)	94	<0.001	7	1.49 (–2.84 to 5.81)	91	<0.001	7	–1.31 (–10.37 to 10.11)	91	<0.001	11	0.96 (–0.29 to 2.22)	89	<0.001	6	–0.01 (–0.22 to 0.20)	96	<0.001
Previous PI (+), Current PI (+)	7	10.91 (0.26–21.56)	97	<0.001	10	9.66 (3.98–15.34)	95	<0.001	9	10.39 (1.64–19.14)	90	<0.001	7	1.47 (–0.54 to 3.49)	94	<0.001	7	0.12 (0.01–0.22)	93	<0.001

Abbreviations: CI, confidence interval; LDL, low‐density lipoprotein; NA, not available; PI, protease inhibitor; TAF, tenofovir alafenamide.

### 3.4 Differences in lipid profile between TAF‐based regimens and TDF‐based regimens

Subsequently, we compared lipid‐profile differences between patients on TAF and TDF regimens (Table 5). After 12 months, TAF versus TDF regimens resulted in significantly higher levels of HDL‐cholesterol (mean difference 2.53 mg/dl; 95% CI: 1.02–4.05), LDL‐cholesterol (10.13 mg/dl; 95% CI: 6.66–13.59), total cholesterol (14.65 mg/dl; 95% CI: 9.99–19.32) and triglycerides (6.88 mg/dl; 95% CI: 0.41–13.35). The forest plots are presented in Figure S2.

**Table 5:** Changes in lipid profile at 1 year after TAF versus TDF treatment

Outcome	No. of studies	Mean difference	95% CI	*I* ^2^	*p* for heterogeneity
HDL‐cholesterol	14	2.53	1.02–4.05	92	<0.01
LDL‐cholesterol	17	10.13	6.66–13.59	89	<0.01
Total cholesterol	18	14.65	9.99–19.32	93	<0.01
Triglycerides	15	6.88	0.41–13.35	90	<0.01
Total cholesterol/HDL‐cholesterol ratio	14	0.11	0.04–0.17	93	<0.01

Abbreviations: CI, confidence interval; HDL, high‐density lipoprotein; LDL, low‐density lipoprotein; TAF, tenofovir alafenamide; TDF, tenofovir disoproxil fumarate.

### 3.5 Lipid‐prolife changes after discontinuation of TAF‐based regimens

Four studies reported lipid profiles after discontinuation of the TAF regimens. We analysed the lipid‐profile changes at 12 months after discontinuation of the TAF regimens (Table 6). Significant decreases in LDL‐cholesterol (mean difference 9.31 mg/dl; 95% CI: 5.36–13.27) and total cholesterol (8.91 mg/dl; 95% CI: 3.88–13.94) were observed 12 months after stopping TAF treatment compared with during TAF treatment. The forest plots are presented in Figure S3.

**Table 6:** Changes in lipid profile 1 year after stopping TAF

Outcome	No. of studies	Mean difference	95% CI	*I* ^2^	*p* for heterogeneity
HDL‐cholesterol	4	–1.86	–3.19 to –0.53	44	0.15
LDL‐cholesterol	4	–9.31	–13.27 to –5.36	60	0.06
Total cholesterol	4	–8.91	–13.94 to –3.88	68	0.02
Triglycerides	4	–9.28	–12.99 to +5.57	0	0.68

Abbreviations: CI, confidence interval; HDL, high‐density lipoprotein; LDL, low‐density lipoprotein; TAF, tenofovir alafenamide.

### 3.6 Changes in bodyweight after TAF use

Twenty‐two studies documented changes in bodyweight after TAF use, revealing an average weight gain of 1.38 kg (95% CI: 0.92–1.84) over the entire observation period (Table 7). Weight gain was more pronounced the longer TAF was used: an increase of 0.64 kg (95% CI: 0.19–1.10) at 6 months, 0.91 kg (95% CI: 0.54–1.27) at 12 months, 1.71 kg (95% CI: 0.64–2.78) at 24 months and 3.42 kg (95% CI: 1.59–5.26) at 36 months. The forest plots are presented in Figure S4.

**Table 7:** Changes in bodyweight (kg) during TAF treatment (vs. baseline)

Outcome	No. of studies	Mean difference	95% CI	*I* ^2^	*p* for heterogeneity
Overall period	22	1.38	0.92–1.84	92	<0.01
6 months	4	0.64	0.19–1.10	67	0.03
12 months	13	0.91	0.54–1.27	75	<0.01
24 months	2	1.71	0.64–2.78	49	0.16
36 months	4	3.42	1.59–5.26	94	<0.01

Abbreviations: CI, confidence interval; TAF, tenofovir alafenamide.

### 3.7 Risk factors for worsening cholesterol in patients using TAF

Finally, a meta‐regression analysis was conducted to identify risk factors associated with worsening cholesterol in patients taking TAF. Our meta‐analysis considered eight risk factors: the proportion of treatment‐naive patients, age, sex, BMI, diabetes, hypertension, prior dyslipidaemia and smoking (Table 8). Our meta‐regression analysis identified several study‐level factors associated with changes in lipid profiles in patients taking TAF. Younger age, male sex and lower baseline BMI were found to be significant risk factors for worsening lipid profiles. Notably, studies with participants having a worse metabolic profile at baseline (higher LDL and total cholesterol) were associated with less worsening of lipids on TAF.

**Table 8:** Meta‐regression analysis of risk factors for worsening lipid profiles in patients treated with TAF for 1 year

Variable	Δ Total cholesterol			Δ LDL‐cholesterol			Δ Triglycerides			Δ HDL‐cholesterol			Δ Total cholesterol/HDL‐cholesterol ratio		
	No.	Coefficient (95% CI)	*p*	No.	Coefficient (95% CI)	*p*	No.	Coefficient (95% CI)	*p*	No.	Coefficient (95% CI)	*p*	No.	Coefficient (95% CI)	*p*
Treatment‐naïve (vs. treatment‐experienced)	40	12.268 (3.670–20.867)	0.005	39	4.643 (–1.185 to 10.472)	0.118	37	9.918 (2.083–17.754)	0.013	36	3.170 (1.562–4.778)	0.001	30	–0.016 (–0.130 to 0.097)	0.774
Age	40	–0.843 (–1.198 to –0.488)	<0.001	39	–0.421 (–0.684 to –0.158)	0.001	37	–0.648 (–1.085 to –0.211)	0.003	36	–0.148 (–0.220 to –0.077)	<0.001	30	0.000 (–0.005 to 0.004)	0.733
Male (%)	40	0.294 (0.057–0.530)	0.014	39	0.211 (0.057–0.364)	0.007	37	0.330 (0.100–0.560)	0.004	36	0.053 (0.008–0.099)	0.020	30	0.001 (–0.001 to 0.004)	0.475
BMI (kg/m^2^)	24	–3.343 (–5.557 to –1.129)	0.003	21	–1.898 (–3.438 to –0.358)	0.015	22	–6.668 (–9.848 to –3.488)	<0.001	21	–0.828 (–1.360 to –0.295)	0.002	15	0.015 (0.000–0.031)	0.044
Diabetes (%)	16	–1.088 (–2.181 to +0.004)	0.051	14	–0.667 (–1.621 to 0.285)	0.170	14	–0.526 (–2.628 to 1.576)	0.623	15	–0.162 (–0.320 to –0.005)	0.042	9	0.025 (0.003–0.047)	0.021
Hypertension (%)	16	–0.586 (–1.023 to –0.149)	0.008	15	–0.405 (–0.736 to –0.075)	0.015	15	–0.611 (–1.364 to 0.141)	0.111	15	–0.083 (–0.136 to –0.030)	0.002	10	0.004 (–0.002 to 0.011)	0.164
Prior dyslipidaemia (%)	13	–0.581 (–1.054 to –0.107)	0.016	12	–0.365 (–0.657 to –0.072)	0.014	14	–1.060 (–1.680 to –0.439)	<0.001	12	–0.105 (–0.174 to –0.037)	0.003	7	0.006 (–0.012 to 0.025)	0.515
Smoking (%)	14	–0.362 (–1.087 to +0.362)	0.327	10	–0.161 (–0.640 to 0.318)	0.510	11	0.033 (–0.683 to 0.749)	0.927	12	–0.040 (–0.122 to 0.041)	0.331	9	–0.004 (–0.009 to 0.000)	0.094
Baseline HDL‐cholesterol level	29	–2.187 (–2.972 to –1.401)	<0.001	26	–1.434 (–2.022 to –0.845)	<0.001	24	–1.845 (–2.779 to –0.911)	<0.001	28	–0.458 (–0.619 to –0.297)	<0.001	20	0.000 (–0.005 to 0.007)	0.871
Baseline LDL‐cholesterol level	29	–0.817 (–1.247 to –0.388)	0.002	28	–0.548 (–0.817 to –0.278)	<0.001	26	–1.084 (–1.530 to –0.639)	<0.001	28	–0.173 (–0.277 to –0.068)	0.001	21	–0.006 (–0.011 to –0.002)	0.001
Baseline total cholesterol level	30	–0.834 (–1.091 to –0.577)	<0.001	26	–0.492 (–0.681 to –0.302)	<0.001	25	–0.809 (–1.144 to –0.474)	<0.001	28	–0.174 (–0.243 to –0.105)	<0.001	20	–0.002 (–0.005 to 0.000)	0.051
Baseline triglyceride level	28	–0.237 (–0.518 to 0.043)	0.097	26	–0.115 (–0.313 to 0.082)	0.251	28	–0.315 (–0.622 to –0.008)	0.044	26	–0.025 (–0.102 to 0.051)	0.513	19	–0.002 (–0.004 to 0.001)	0.111

Abbreviations: BMI, body mass index; CI, confidence interval; LDL, low‐density lipoprotein; TAF, tenofovir alafenamide.

## 4 DISCUSSION

This meta‐analysis consolidates existing evidence on the impact of TAF on lipid metabolism in individuals undergoing HIV treatment. Consistent with the primary objective, our findings indicate significantly altered lipid profiles post‐TAF treatment, characterized by increased levels of HDL‐cholesterol, LDL‐cholesterol, total cholesterol and triglycerides. Notably, discontinuation of TAF was associated with a marked decrease in LDL‐cholesterol and total cholesterol, suggesting a direct impact of TAF on lipid metabolism, although the influence of other factors alongside TAF discontinuation cannot be ruled out, especially since the four studies included in this analysis were not randomized.

Our study consolidates existing evidence on the impact of TAF on lipid metabolism in individuals undergoing HIV treatment. Consistent with the primary objective, our findings indicate significantly altered lipid profiles post‐TAF treatment, characterized by increased levels of HDL‐cholesterol, LDL‐cholesterol, total cholesterol and triglycerides. Notably, discontinuation of TAF was associated with a marked decrease in LDL‐cholesterol and total cholesterol, suggesting a direct impact of TAF on lipid metabolism. The observed improvements in lipid profiles upon discontinuation of TAF provide compelling evidence that TAF exacerbates hyperlipidaemia, which can be reversed by switching to alternative antiretroviral therapies. Given the significant increases in LDL and total cholesterol observed in our analysis, the potential increase in CVD risk necessitates vigilant metabolic health monitoring in patients on TAF.

Interestingly, while elevated HDL‐cholesterol could be interpreted as a positive outcome, given the protective role of HDL‐cholesterol against CVD, the simultaneous increase in LDL‐cholesterol and triglycerides complicates this narrative. While our study focused primarily on individual lipid parameters, the net impact of TAF on the total cholesterol to total cholesterol/HDL‐C ratio is also important to consider. Despite observing increases in both total cholesterol and HDL, the net change in the total cholesterol/HDL‐C ratio remained relatively even. However, the absolute increases in LDL and total cholesterol suggest an unfavourable cardiovascular profile, as elevated levels of these lipids are strongly associated with increased CVD risk. These changes align with research suggesting that certain ART agents, particularly regimens including TAF, may shift lipid profiles towards patterns associated with increased cardiovascular risk. Among ART agents, protease inhibitors and elvitegravir are known to require caution in patients with dyslipidaemia, and TAF should be added to this list.

Various antiretroviral drugs affect lipid profiles: for example, it is well known that protease inhibitors such as ritonavir, efavirenz and elvitegravir worsen lipid profiles, mostly affecting triglyceride levels [75, 76]. We tried to capture the lipid‐altering effects of antiretroviral drugs other than TAF. Although detailed data on all antiretroviral regimens were not available in the 39 articles included, efavirenz and elvitegravir were used in only a negligible proportion of the study populations. Thus, we evaluated subgroups according to the use of protease inhibitors before and during TAF use. After the discontinuation of protease inhibitors, our data suggested a decreasing trend in triglyceride levels, even after the initiation of TAF. However, the estimated change of –1.31 (95% CI –10.37 to 10.11) was not statistically significant. TAF‐induced changes in LDL‐cholesterol levels were unaffected by the discontinuation of protease inhibitors. In contrast to other drugs, TDF has a favourable effect on lipid profiles. We identified that 41.5% of the total study population switched from TDF to TAF, contributing to more dramatic changes in lipid levels. Interestingly, the potential mechanism behind TDF's beneficial impact on lipid profiles might be linked to its effect on nutrient absorption. TDF has been hypothesized to cause damage to enterocytes, which could impair nutrient absorption and suppress weight gain, subsequently affecting lipid levels [77]. This hypothesis suggests that the higher lipid levels observed with TAF use may partly reflect the resolution of TDF‐induced derangements.

Besides the use of people living with or persons living with HIV, TAF is also used to treat hepatitis B virus (HBV) infection. In patients with HBV infection, worsening dyslipidaemia after TAF use was reported in a meta‐analysis [8]. The exact reasons behind the less advantageous impact of TAF on lipid profiles remain unclear. However, based on study outcomes, we can suggest two plausible explanations. Firstly, TAF use correlates with weight gain, which can worsen metabolic conditions; indeed, in our study, body weight increased as the duration of TAF use increased. Studies from various regions reported immediate weight gain after switching to TAF in people living with or persons living with HIV. Such weight gain is likely to aggravate dyslipidaemia and metabolic disorders, linking TAF use to worsening lipid profiles. Secondly, TAF may directly worsen lipid profiles, whereas TDF appears to have a beneficial effect [78, 79, 80]. TDF, compared to TAF, presents a higher concentration of the active drug tenofovir in the bloodstream, which could positively influence lipid levels [81, 82]. Additionally, laboratory research indicates that TDF may reduce cholesterol in hepatocyte supernatant by stimulating the peroxisome proliferator‐activated receptor alpha pathway [83]. Such stimulation promotes the expression of genes like carnitine palmitoyltransferase 1 and *CD36*, with the latter facilitating the uptake of oxidized LDL‐cholesterol, HDL‐cholesterol and free fatty acids by the liver, thus offering a protective effect on lipids [84, 85, 86, 87]. Nonetheless, these mechanisms have not been definitively proven in human studies, underscoring the need for further investigation.

Our meta‐regression analysis revealed that younger age, male sex and lower baseline BMI were significant predictors of worsening lipid profiles in patients on TAF. Interestingly, a worse metabolic profile at baseline was associated with less worsening of lipids on TAF. This finding suggests that patients with higher baseline cholesterol levels may experience smaller increases in lipid levels during TAF treatment, aligning with some previous studies [8, 88]. Currently, the clinical implication of this finding is not clear. Further study is needed to identify whether TAF additionally increases cardiovascular risk in patients who already have dyslipidaemia. Basically, TAF could be switched to alternative antiretrovirals in patients with higher cardiovascular risks to avoid any potential harm.

The geographical distribution of the studies included in our meta‐analysis predominantly represents high‐income countries, such as the United States and European nations. This distribution suggests that the findings may be influenced by the healthcare infrastructure and resources available in these regions, which can affect the generalizability of the results to lower‐income countries. In high‐income countries, the monitoring and management of lipid profiles in people living with or persons living with HIV might be more systematic and accessible, potentially leading to better detection and treatment of dyslipidaemia. However, in low‐ and middle‐income countries, where fewer studies were conducted, healthcare resources may be limited, and the impact of TAF on lipid metabolism could be underreported or inadequately managed.

Our study has several limitations. Firstly, the heterogeneity among included studies, regarding study design, population demographics and treatment duration, may influence the generalizability of the overall findings. The potential for selection bias exists as we included only studies that provided exact numerical values for cholesterol level changes, excluding those that reported changes in percentages or proportions. Secondly, the main limitation of our study is that the impact of lipid changes caused by TAF on the long‐term cardiovascular outcome could not be determined by our study. Only four studies presented the other CVD risk factors and data on the long‐term cardiovascular outcome lacked in other studies. Additionally, even these four studies used different parameters to present CVD risk, making it difficult to integrate the results into meta‐analysis. However, we want to focus on the fact that the significant increases in LDL‐cholesterol and total cholesterol observed in our analysis could be associated with potential increases or with a potential increase in CVD risk. Although the direct association of TAF use with CVD event is beyond the scope of our study, an average increase of 12.31 mg/dl LDL‐cholesterol and 18.86 mg/dl in total cholesterol after TAF initiation could potentially increase CVD risk, particularly in individuals with additional risk factors such as hypertension, diabetes or smoking. Further studies should focus on long‐term outcomes of TAF treatment, especially concerning cardiovascular events. Although our analyses focused on antiretroviral drugs well known for affecting lipid profiles, some unexpected bias may have resulted from the effect of companion drugs. Thirdly, it was challenging to accurately estimate the potential effects of individual antiretroviral drugs other than TAF. A wide variety of antiretroviral regimens were used in conjunction with TAF in each study and some regimens were included in only one or two studies.

## 5 CONCLUSIONS

In conclusion, while TAF represents a significant advance in HIV treatment, its impact on lipid metabolism warrants careful consideration. The findings from this meta‐analysis highlight the necessity for comprehensive metabolic monitoring and management strategies for patients on TAF, to mitigate the increased risk of CVD. As the landscape of HIV treatment continues to evolve, so too must our understanding of the long‐term implications of ART on overall health.

## COMPETING INTERESTS

The authors have no relevant financial or non‐financial interests to disclose.

## AUTHORS’ CONTRIBUTIONS

Study concept and design: J‐JY and MJK; Provision of study materials or patients: J‐JY and EAJ; Collection and assembly of data: SGK and YSK; Data analysis and interpretation J‐JY and MJK; Manuscript writing: J‐JY; Final approval of manuscript: All authors.

## FUNDING

This work was supported by the Soonchunhyang University Research Fund.

## Supporting information



Figure S1. Forest plots and funnel plots of Table 2
Figure S2. Forest plots and funnel plots of Table 5
Figure S3. Forest plots and funnel plots of Table 6
Figure S4. Forest plots and funnel plots of Table 7

## Data Availability

The datasets generated during and/or analysed during the current study are available from the corresponding author upon reasonable request.
