# Supplementary material for: Risk of dyslipidaemia in people living with HIV who are taking tenofovir alafenamide: a systematic review and meta‐analysis
Source: J Int AIDS Soc. 2024 Sep 20;27(9):e26358. doi: 10.1002/jia2.26358 (PMC11413498; doi:10.1002/jia2.26358)
Supplement: Supplementary file 1 — Figure S1. Forest plots and funnel plots of Table 2 Figure S2. Forest plots and funnel plots of Table 5 Figure S3. Forest plots and funnel plots of Table 6 Figure S4. Forest plots and funnel plots of Table 7 [file JIA2-27-e26358-s001.docx]

**Supplementary Material**

**Table of Contents**

**I. Supplementary Materials and Methods**………………………………………..…..….2

Search strategy and results

Risk of bias

**II. Supplementary Figures....**……………...……………………………….…………..….11

**I. Supplementary Materials and Methods**

**1. Search strategy and results**

**(1) Summary of search results**

| **No.** | **DB** | **Results** | **Duplication** |
| --- | --- | --- | --- |
| 1 | PubMed (Medline) | 358 | 798 |
| 2 | EMBASE | 1,801 |  |
| 3 | Cochrane Library | 346 |  |
| 4 | Web of Science | 307 |  |
| 5 | KoreaMed | 2 |  |
| Number of Search Results (including duplication) | | 2,814 |  |
| **Number of Search Results (without duplication)** | | **2,016** |  |

**(2) Keywords**

| **PICO** | **Fields** | **Keywords** | **Remarks** |
| --- | --- | --- | --- |
| **P** | MeSH | HIV | **A** |
|  | MeSH | Human Immunodeficiency Virus* |  |
|  | TIAB | Human T Cell Lymphotropic Virus* | **B** |
|  | TIAB | Human T Lymphotropic Virus* |  |
|  | TIAB | Human T Cell Leukemia Virus* |  |
|  | TIAB | Human T Leukemia Virus* |  |
|  | TIAB | Lymphadenopathy-Associated Virus* |  |
|  | TIAB | AIDS |  |
|  | TIAB | Acquired Immune Deficiency Syndrome* |  |
|  | TIAB | Acquired Immunodeficiency Syndrome* |  |
|  | TIAB | HIV |  |
|  | TIAB | HTLV-II |  |
|  |  | **A OR B** | **C** |
| **I** | MeSH | Tenofovir | **D** |
|  | Supplementary Concept | Tenofovir alafenamide | **E** |
|  | TIAB | Tenofovir | **F** |
|  | TIAB | Tenofovir alafenamide |  |
|  | TIAB | Tenofovir-A |  |
|  | TIAB | Vemlidy |  |
|  | TIAB | GS-7340 |  |
|  | TIAB | GS-734003 |  |
|  | TIAB | TAF |  |
|  | TIAB | Tenofovir disoproxil |  |
|  | TIAB | Tenofovir-DF |  |
|  | TIAB | 9-(2-Phosphonylmethoxypropyl)adenine |  |
|  | TIAB | 9-(2-Phosphonomethoxypropyl)adenine |  |
|  | TIAB | (R)-9-(2-phosphonylmethoxypropyl)adenine |  |
|  | TIAB | Viread |  |
|  | TIAB | PMPA |  |
|  | TIAB | TDF |  |
|  |  | **D OR E OR F** | **G** |
| **O** | MeSH | Dyslipidemias | **H** |
|  | MeSH | Hyperlipidemias |  |
|  | MeSH | Hypercholesterolemia |  |
|  | MeSH | Cholesterol |  |
|  | MeSH | Cholesterol, HDL |  |
|  | MeSH | Lipoproteins, HDL |  |
|  | MeSH | Cholesterol, LDL |  |
|  | MeSH | Lipoproteins, LDL |  |
|  | MeSH | Triglycerides |  |
|  | Supplementary Concept | HDL-triglyceride | **I** |
|  | Supplementary Concept | Low density lipoprotein triglyceride |  |
|  | Supplementary Concept | Very low density lipoprotein triglyceride |  |
|  | TIAB | Dyslipidemia* | **J** |
|  | TIAB | Dyslipoproteinemia* |  |
|  | TIAB | Hyperlipidemia* |  |
|  | TIAB | Hyperlipemia* |  |
|  | TIAB | Lipidemia* |  |
|  | TIAB | Lipemia* |  |
|  | TIAB | Hypercholesterolemia* |  |
|  | TIAB | Hypercholesteremia* |  |
|  | TIAB | Cholesterol* |  |
|  | TIAB | Epicholesterol* |  |
|  | TIAB | Cholesterin* |  |
|  | TIAB | High-density lipoprotein* |  |
|  | TIAB | HDL* |  |
|  | TIAB | alpha-lipoprotein* |  |
|  | TIAB | α-lipoprotein* |  |
|  | TIAB | Heavy lipoprotein* |  |
|  | TIAB | alpha-1 lipoprotein* |  |
|  | TIAB | α-1 lipoprotein* |  |
|  | TIAB | beta-lipoprotein* |  |
|  | TIAB | β-lipoprotein* |  |
|  | TIAB | Low-density lipoprotein* |  |
|  | TIAB | LDL* |  |
|  | TIAB | Triglyceride* |  |
|  | TIAB | Triacylglycerol* |  |
|  | TIAB | Triacylglyceride* |  |
|  | TIAB | Lipid profile* |  |
|  | TIAB | High lipid* |  |
|  | TIAB | Low lipid* |  |
|  |  | **H OR I OR J** | **K** |
|  |  | **C AND G AND K** |  |

**(3) Search strategy**

| **DB** | **Search Strategy** |
| --- | --- |
| **PubMed** | ("HIV"[MeSH Terms] OR "hiv infection*"[MeSH Terms] OR ("human immunodeficiency virus*"[Title/Abstract] OR "human t cell lymphotropic virus*"[Title/Abstract] OR "human t lymphotropic virus*"[Title/Abstract] OR "human t cell leukemia virus*"[Title/Abstract] OR "human t leukemia virus*"[Title/Abstract] OR "lymphadenopathy associated virus*"[Title/Abstract] OR "AIDS"[Title/Abstract] OR "acquired immune deficiency syndrome*"[Title/Abstract] OR "acquired immunodeficiency syndrome*"[Title/Abstract] OR "HIV"[Title/Abstract] OR "HTLV-II"[Title/Abstract])) AND ("Tenofovir"[MeSH Terms] OR "Tenofovir alafenamide"[Supplementary Concept] OR ("Tenofovir"[Title/Abstract] OR "Tenofovir alafenamide"[Title/Abstract] OR "Tenofovir-A"[Title/Abstract] OR "Vemlidy"[Title/Abstract] OR "GS-7340"[Title/Abstract] OR "TAF"[Title/Abstract] OR "Tenofovir disoproxil"[Title/Abstract] OR "Tenofovir-DF"[Title/Abstract] OR "9 2 phosphonylmethoxypropyl adenine"[Title/Abstract] OR "9 2 phosphonomethoxypropyl adenine"[Title/Abstract] OR "r 9 2 phosphonylmethoxypropyl adenine"[Title/Abstract] OR "Viread"[Title/Abstract] OR "PMPA"[Title/Abstract] OR "TDF"[Title/Abstract])) AND ("dyslipidemias"[MeSH Terms] OR "hyperlipidemias"[MeSH Terms] OR "hypercholesterolemia"[MeSH Terms] OR "cholesterol"[MeSH Terms] OR "cholesterol, hdl"[MeSH Terms] OR "lipoproteins, hdl"[MeSH Terms] OR "cholesterol, ldl"[MeSH Terms] OR "lipoproteins, ldl"[MeSH Terms] OR "triglycerides"[MeSH Terms] OR ("hdl triglyceride"[Supplementary Concept] OR "low density lipoprotein triglyceride"[Supplementary Concept] OR "very low density lipoprotein triglyceride"[Supplementary Concept]) OR ("dyslipidemia*"[Title/Abstract] OR "dyslipoproteinemia*"[Title/Abstract] OR "hyperlipidemia*"[Title/Abstract] OR "hyperlipemia*"[Title/Abstract] OR "lipidemia*"[Title/Abstract] OR "lipemia*"[Title/Abstract] OR "hypercholesterolemia*"[Title/Abstract] OR "hypercholesteremia*"[Title/Abstract] OR "cholesterol*"[Title/Abstract] OR "epicholesterol*"[Title/Abstract] OR "cholesterin*"[Title/Abstract] OR "high density lipoprotein*"[Title/Abstract] OR "hdl"[Title/Abstract] OR "alpha lipoprotein*"[Title/Abstract] OR "alpha lipoprotein*"[Title/Abstract] OR "heavy lipoprotein*"[Title/Abstract] OR "alpha 1 lipoprotein*"[Title/Abstract] OR "alpha 1 lipoprotein*"[Title/Abstract] OR "beta lipoprotein*"[Title/Abstract] OR "beta lipoprotein*"[Title/Abstract] OR "low density lipoprotein*"[Title/Abstract] OR "ldl"[Title/Abstract] OR "triglyceride*"[Title/Abstract] OR "triacylglycerol*"[Title/Abstract] OR "triacylglyceride*"[Title/Abstract] OR "lipid profile*"[Title/Abstract] OR "high lipid*"[Title/Abstract] OR "low lipid*"[Title/Abstract])) |
| **EMBASE** | (('human immunodeficiency virus infection'/exp OR 'human immunodeficiency virus'/exp OR 'human immunodeficiency virus 2'/exp OR 'human immunodeficiency virus 1'/exp OR 'acquired immune deficiency syndrome'/exp OR 'aids patient'/exp OR 'human t-lymphotropic virus 2'/exp) OR ('human immunodeficiency virus*':ab,ti OR 'human t cell lymphotropic virus*':ab,ti OR 'human t-cell leukemia virus*':ab,ti OR 'human t lymphotropic virus*':ab,ti OR 'human t leukemia virus*':ab,ti OR 'lymphadenopathy-associated virus*':ab,ti OR 'aids':ab,ti OR 'acquired immune deficiency syndrome*':ab,ti OR 'acquired immunodeficiency syndrome*':ab,ti OR 'hiv':ab,ti OR 'htlv-ii':ab,ti)) AND (('tenofovir'/exp OR 'tenofovir alafenamide'/exp OR 'tenofovir disoproxil'/exp) OR ('tenofovir':ab,ti OR 'tenofovir alafenamide':ab,ti OR 'tenofovir-a':ab,ti OR 'vemlidy':ab,ti OR 'gs-7340':ab,ti OR 'gs-734003':ab,ti OR 'taf':ab,ti OR 'tenofovir disoproxil':ab,ti OR 'tenofovir-df':ab,ti OR '9-(2-phosphonylmethoxypropyl)adenine':ab,ti OR '9-(2-phosphonomethoxypropyl)adenine':ab,ti OR '(r)-9-(2-phosphonylmethoxypropyl)adenine':ab,ti OR 'viread':ab,ti OR 'pmpa':ab,ti OR 'tdf':ab,ti)) AND (('dyslipidemia'/exp OR 'hyperlipidemia'/exp OR 'hypercholesterolemia'/exp OR 'cholesterol'/exp OR 'high density lipoprotein cholesterol'/exp OR 'high density lipoprotein'/exp OR 'low density lipoprotein cholesterol'/exp OR 'low density lipoprotein'/exp OR 'triacylglycerol'/exp OR 'hdl triglyceride'/exp OR 'dyslipoproteinemia'/exp OR 'lipoprotein'/exp) OR ('dyslipidemia*':ab,ti OR 'dyslipoproteinemia*':ab,ti OR 'hyperlipidemia*':ab,ti OR 'hyperlipemia*':ab,ti OR 'lipidemia*':ab,ti OR 'lipemia*':ab,ti OR 'hypercholesterolemia*':ab,ti OR 'hypercholesteremia*':ab,ti OR 'cholesterol*':ab,ti OR 'epicholesterol*':ab,ti OR 'cholesterin*':ab,ti OR 'high-density lipoprotein*':ab,ti OR 'hdl*':ab,ti OR 'alpha-lipoprotein*':ab,ti OR 'α-lipoprotein*':ab,ti OR 'heavy lipoprotein*':ab,ti OR 'alpha-1 lipoprotein*':ab,ti OR 'α-1 lipoprotein*':ab,ti OR 'beta-lipoprotein*':ab,ti OR 'β-lipoprotein*':ab,ti OR 'low-density lipoprotein*':ab,ti OR 'ldl*':ab,ti OR 'triglyceride*':ab,ti OR 'triacylglycerol*':ab,ti OR 'triacylglyceride*':ab,ti OR 'lipid profile*':ab,ti OR 'high lipid*':ab,ti OR 'low lipid*':ab,ti)) |
| **Cochrane** | (((MeSH descriptor: [HIV] explode all trees OR MeSH descriptor: [HIV Infections] in all MeSH products) OR ((human immunodeficiency virus*):ti,ab,kw OR (human t cell lymphotropic virus*):ti,ab,kw OR (human t lymphotropic virus*):ti,ab,kw OR (human t cell leukemia virus*):ti,ab,kw OR (human t leukemia virus*):ti,ab,kw OR (lymphadenopathy associated virus*):ti,ab,kw OR (AIDS):ti,ab,kw OR (acquired immune deficiency syndrome*):ti,ab,kw OR (acquired immunodeficiency syndrome*):ti,ab,kw OR (HIV):ti,ab,kw OR (HTLV-II):ti,ab,kw)) AND ((MeSH descriptor: [Tenofovir] explode all trees) OR ((Tenofovir):ti,ab,kw OR (Tenofovir alafenamide):ti,ab,kw OR (Tenofovir-A):ti,ab,kw OR (Vemlidy):ti,ab,kw OR (GS-7340):ti,ab,kw OR (TAF):ti,ab,kw OR (Tenofovir disoproxil):ti,ab,kw OR (Tenofovir-DF):ti,ab,kw OR (9 2 phosphonylmethoxypropyl adenine):ti,ab,kw OR (9 2 phosphonomethoxypropyl adenine):ti,ab,kw OR (r 9 2 phosphonylmethoxypropyl adenine):ti,ab,kw OR (Viread):ti,ab,kw OR (PMPA):ti,ab,kw OR (TDF):ti,ab,kw)) AND ((MeSH descriptor: [Dyslipidemias] explode all trees OR MeSH descriptor: [Hyperlipidemias] explode all trees OR MeSH descriptor: [Hypercholesterolemia] explode all trees OR MeSH descriptor: [Cholesterol] explode all trees OR MeSH descriptor: [Cholesterol, HDL] explode all trees OR MeSH descriptor: [Lipoproteins, HDL] explode all trees OR MeSH descriptor: [Cholesterol, LDL] explode all trees OR MeSH descriptor: [Lipoproteins, LDL] explode all trees OR MeSH descriptor: [Triglycerides] explode all trees) OR ((dyslipidemia*):ti,ab,kw OR (dyslipoproteinemia*):ti,ab,kw OR (hyperlipidemia*):ti,ab,kw OR (hyperlipemia*):ti,ab,kw OR (lipidemia*):ti,ab,kw OR (lipemia*):ti,ab,kw OR (hypercholesterolemia*):ti,ab,kw OR (hypercholesteremia*):ti,ab,kw OR (cholesterol*):ti,ab,kw OR (epicholesterol*):ti,ab,kw OR (cholesterin*):ti,ab,kw OR (high density lipoprotein*):ti,ab,kw OR (hdl):ti,ab,kw OR (alpha lipoprotein*):ti,ab,kw OR (α-lipoprotein*):ti,ab,kw OR (heavy lipoprotein*):ti,ab,kw OR (alpha 1 lipoprotein*):ti,ab,kw OR (α-1 lipoprotein*):ti,ab,kw OR (beta lipoprotein*):ti,ab,kw OR (β-lipoprotein*):ti,ab,kw OR (low density lipoprotein*):ti,ab,kw OR (ldl):ti,ab,kw OR (triglyceride*):ti,ab,kw OR (triacylglycerol*):ti,ab,kw OR (triacylglyceride*):ti,ab,kw OR (lipid profile*):ti,ab,kw OR (high lipid*):ti,ab,kw OR (low lipid*):ti,ab,kw))) NOT (MeSH descriptor: [Animals] this term only NOT (MeSH descriptor: [Animals] this term only AND MeSH descriptor: [Humans] this term only)) |
| **Web of Science** | (TI=("Human Immunodeficiency Virus*" OR "Human T Cell Lymphotropic Virus*" OR "Human T Lymphotropic Virus*" OR "Human T Cell Leukemia Virus*" OR "Human T Leukemia Virus*" OR "Lymphadenopathy-Associated Virus*" OR "AIDS" OR "Acquired Immune Deficiency Syndrome*" OR "Acquired Immunodeficiency Syndrome*" OR "HIV" OR "HTLV-II") OR AB=("Human Immunodeficiency Virus*" OR "Human T Cell Lymphotropic Virus*" OR "Human T Lymphotropic Virus*" OR "Human T Cell Leukemia Virus*" OR "Human T Leukemia Virus*" OR "Lymphadenopathy-Associated Virus*" OR "AIDS" OR "Acquired Immune Deficiency Syndrome*" OR "Acquired Immunodeficiency Syndrome*" OR "HIV" OR "HTLV-II")) AND (TI=(“Tenofovir” OR “Tenofovir alafenamide” OR “Tenofovir-A” OR “Vemlidy” OR “GS-7340” OR “GS-734003” OR “TAF” OR “Tenofovir disoproxil” OR “Tenofovir-DF” OR “9-(2-Phosphonylmethoxypropyl)adenine” OR “9-(2-Phosphonomethoxypropyl)adenine” OR “(R)-9-(2-phosphonylmethoxypropyl)adenine” OR “Viread” OR “PMPA” OR “TDF”) OR AB=(“Tenofovir” OR “Tenofovir alafenamide” OR “Tenofovir-A” OR “Vemlidy” OR “GS-7340” OR “GS-734003” OR “TAF” OR “Tenofovir disoproxil” OR “Tenofovir-DF” OR “9-(2-Phosphonylmethoxypropyl)adenine” OR “9-(2-Phosphonomethoxypropyl)adenine” OR “(R)-9-(2-phosphonylmethoxypropyl)adenine” OR “Viread” OR “PMPA” OR “TDF”)) AND (TI=(“Dyslipidemia*” OR “Dyslipoproteinemia*” OR “Hyperlipidemia*” OR “Hyperlipemia*” OR “Lipidemia*” OR “Lipemia*” OR “Hypercholesterolemia*” OR “Hypercholesteremia*” OR “Cholesterol*” OR “Epicholesterol*” OR “Cholesterin*” OR “High-density lipoprotein*” OR “HDL*” OR “alpha-lipoprotein*” OR “α-lipoprotein*” OR “Heavy lipoprotein*” OR “alpha-1 lipoprotein*” OR “α-1 lipoprotein*” OR “beta-lipoprotein*” OR “β-lipoprotein*” OR “Low-density lipoprotein*” OR “LDL*” OR “Triglyceride*” OR “Triacylglycerol*” OR “Triacylglyceride*” OR “Lipid profile*” OR “High lipid*” OR “Low lipid*”) OR AB=(“Dyslipidemia*” OR “Dyslipoproteinemia*” OR “Hyperlipidemia*” OR “Hyperlipemia*” OR “Lipidemia*” OR “Lipemia*” OR “Hypercholesterolemia*” OR “Hypercholesteremia*” OR “Cholesterol*” OR “Epicholesterol*” OR “Cholesterin*” OR “High-density lipoprotein*” OR “HDL*” OR “alpha-lipoprotein*” OR “α-lipoprotein*” OR “Heavy lipoprotein*” OR “alpha-1 lipoprotein*” OR “α-1 lipoprotein*” OR “beta-lipoprotein*” OR “β-lipoprotein*” OR “Low-density lipoprotein*” OR “LDL*” OR “Triglyceride*” OR “Triacylglycerol*” OR “Triacylglyceride*” OR “Lipid profile*” OR “High lipid*” OR “Low lipid*”)) |
| **KoreaMed** | (((("HIV"[MH])) OR ("HIV Infections"[MH])) OR (((((((("Human Immunodeficiency Virus"[ALL])) OR ("Human T Lymphotropic Virus"[ALL])) OR ("AIDS"[ALL])) OR ("Acquired Immune Deficiency Syndrome"[ALL])) OR ("Acquired Immunodeficiency Syndrome"[ALL])) OR ("HIV"[ALL])) OR ("HTLV-II"[ALL]))) AND ((Tenofovir[MH]) OR (((((("Tenofovir"[ALL])) OR ("GS-7340"[ALL])) OR ("TAF"[ALL])) OR ("Viread"[ALL])) OR ("TDF"[ALL]))) AND ((((((((((("Dyslipidemias"[MH])) OR ("Hyperlipidemias"[MH])) OR ("Hypercholesterolemia"[MH])) OR ("Cholesterol"[MH])) OR ("Cholesterol, HDL"[MH])) OR ("Lipoproteins, HDL"[MH])) OR ("Cholesterol, LDL"[MH])) OR ("Lipoproteins, LDL"[MH])) OR ("Triglycerides"[MH])) OR (((((((((((((((((((((("Dyslipidemia"[ALL])) OR ("Dyslipoproteinemia"[ALL])) OR ("Hyperlipidemia"[ALL])) OR ("Hyperlipemia"[ALL])) OR ("Lipidemia"[ALL])) OR ("Lipemia"[ALL])) OR ("Hypercholesterolemia"[ALL])) OR ("Hypercholesterolemia"[ALL])) OR ("Cholesterol"[ALL])) OR ("High-density lipoprotein"[ALL])) OR ("HDL"[ALL])) OR ("alpha-lipoprotein"[ALL])) OR ("beta-lipoprotein"[ALL])) OR ("Low-density lipoprotein"[ALL])) OR ("LDL"[ALL])) OR ("Triglyceride"[ALL])) OR ("Triacylglycerol"[ALL])) OR ("Triacylglyceride"[ALL])) OR ("Lipid profile"[ALL])) OR ("High lipid"[ALL])) OR ("Low lipid"[ALL]))) NOT ((Animals[MH]) NOT ((Animals[MH]) AND (Humans[MH]))) |

**2. Risk of bias**

1) Randomized controlled study

**
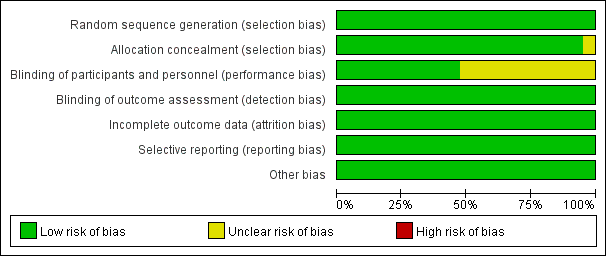
**

**
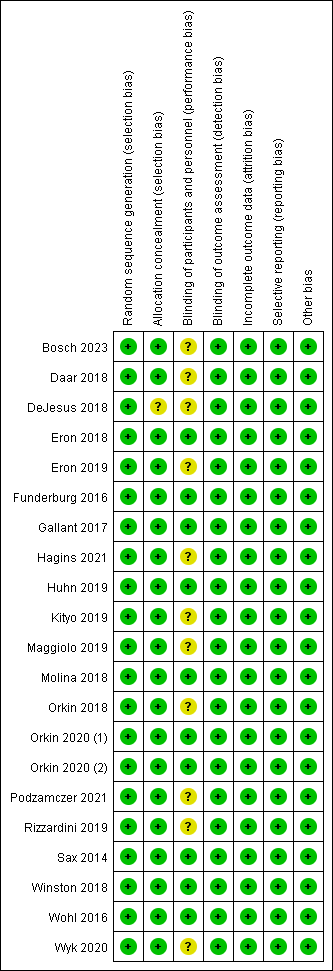
**

2) Non-randomized controlled study including cohort studies


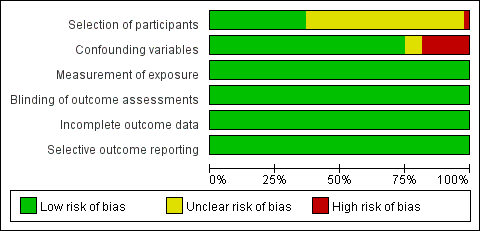


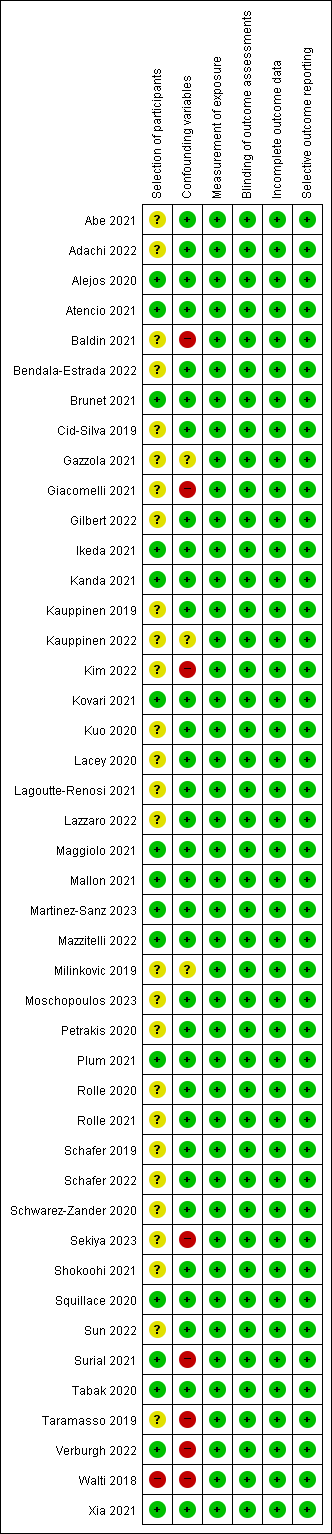


**II. Supplementary Figures**

**Supplementary Figure 1. Forest plots and funnel plots of Table 2**

|  | Forest plots | Funnel Plots |
| --- | --- | --- |
| HDL  3-mo | 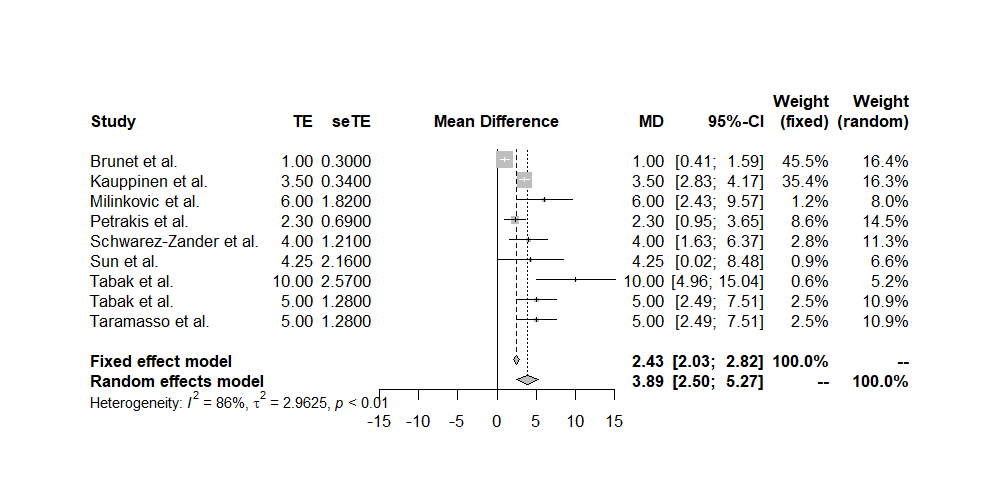 | 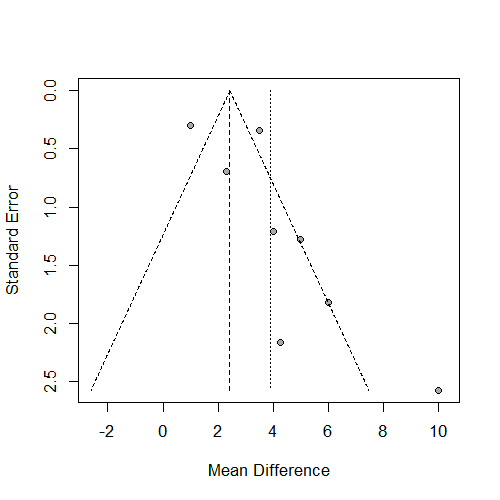 |
| HDL  6-mo | 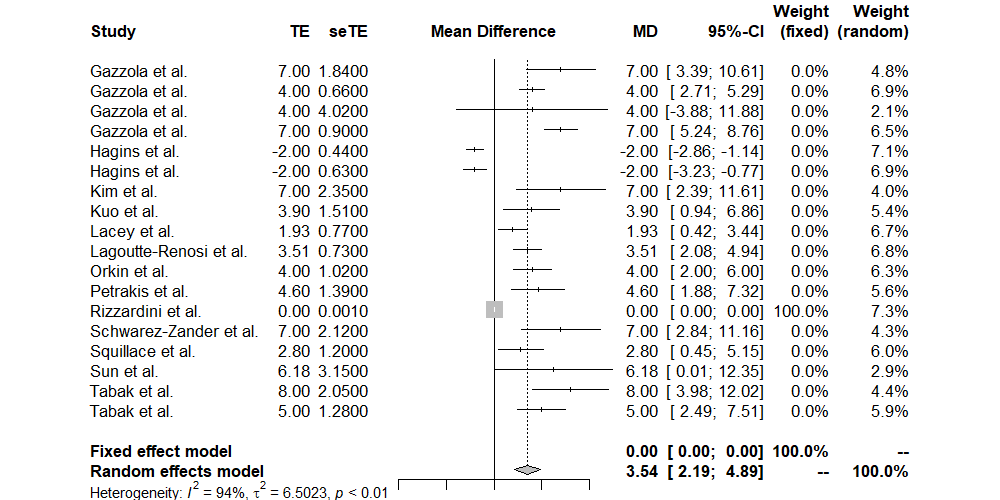 | 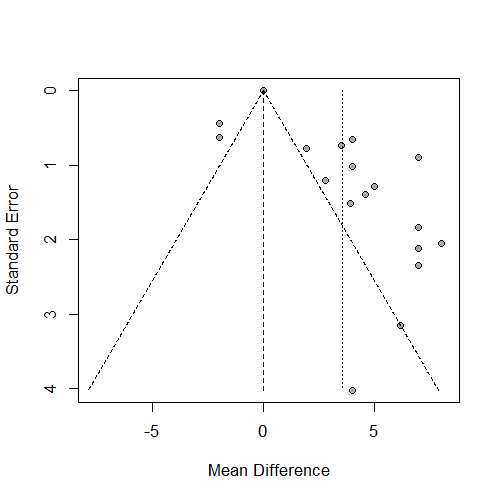 |
| HDL  12-mo | 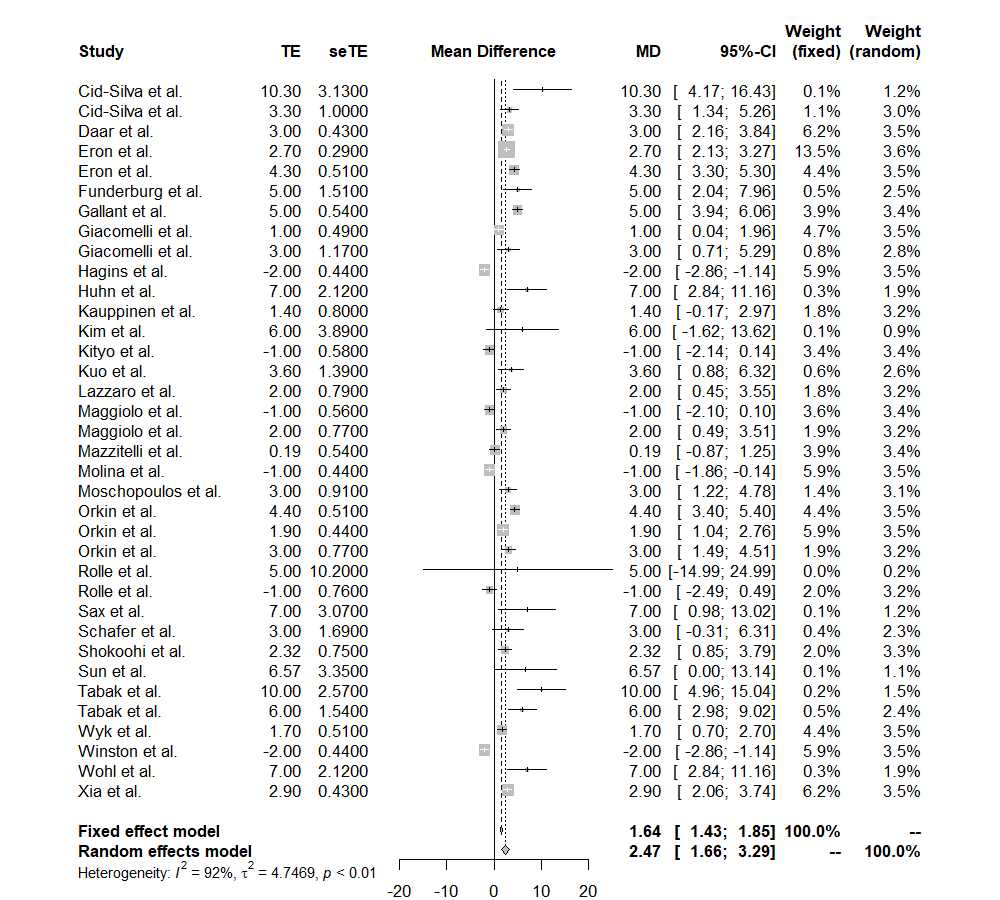 | 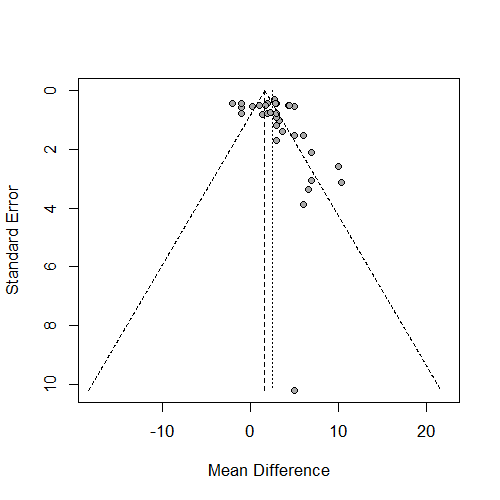 |
| HDL  24-mo | 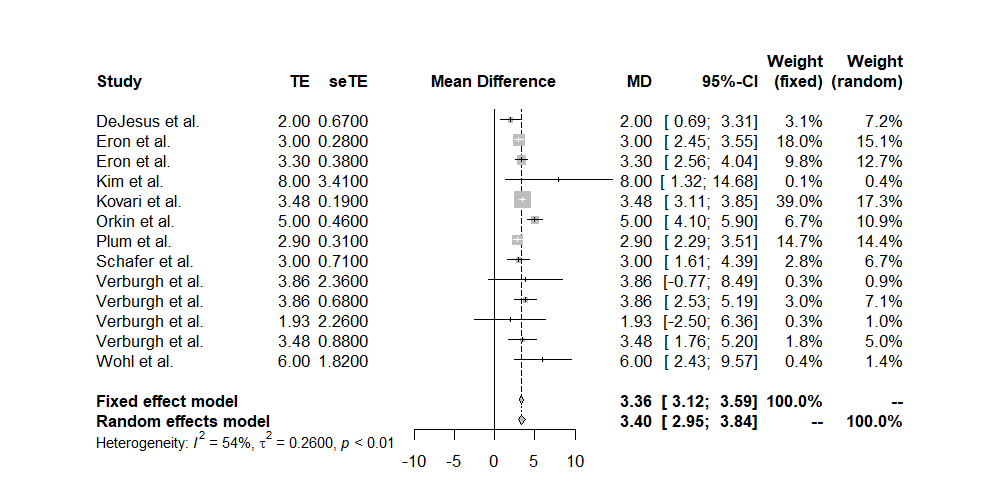 | 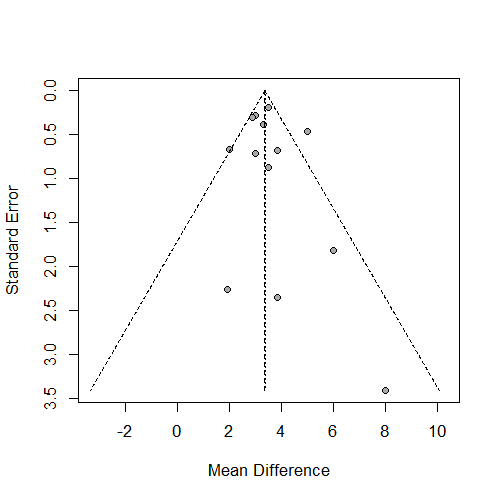 |
| HDL  36-mo | 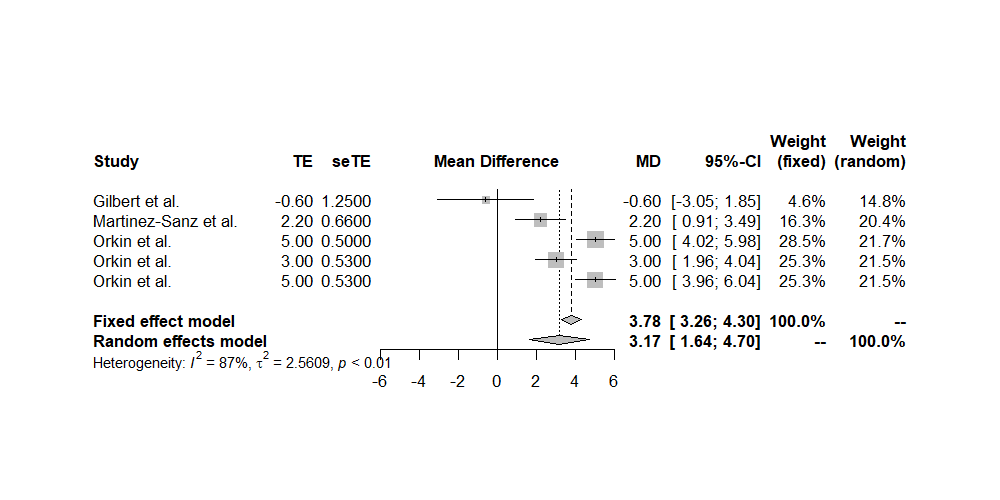 | 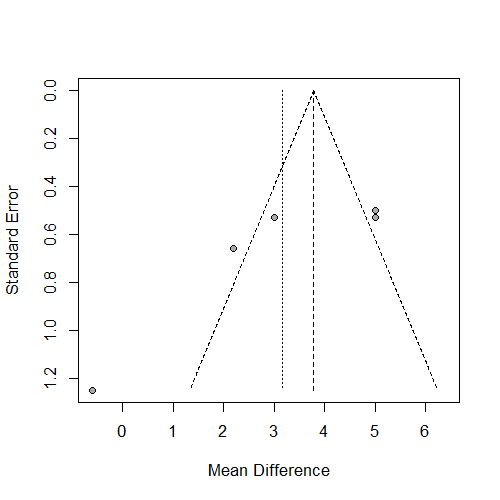 |
| LDL  3-mo | 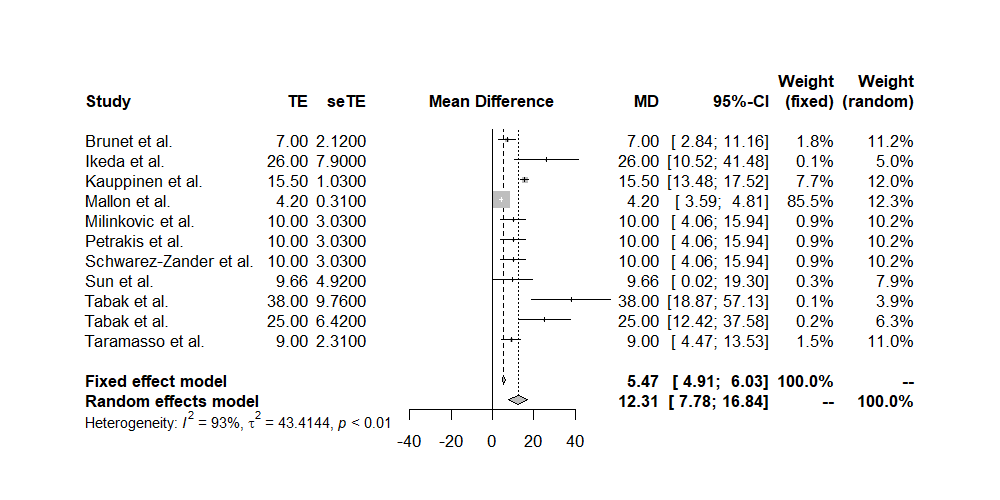 | 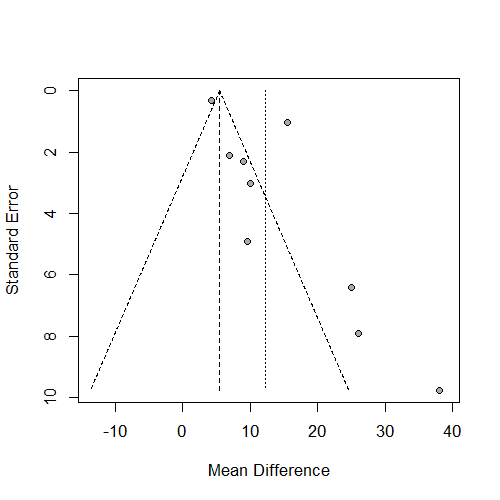 |
| LDL  6-mo | 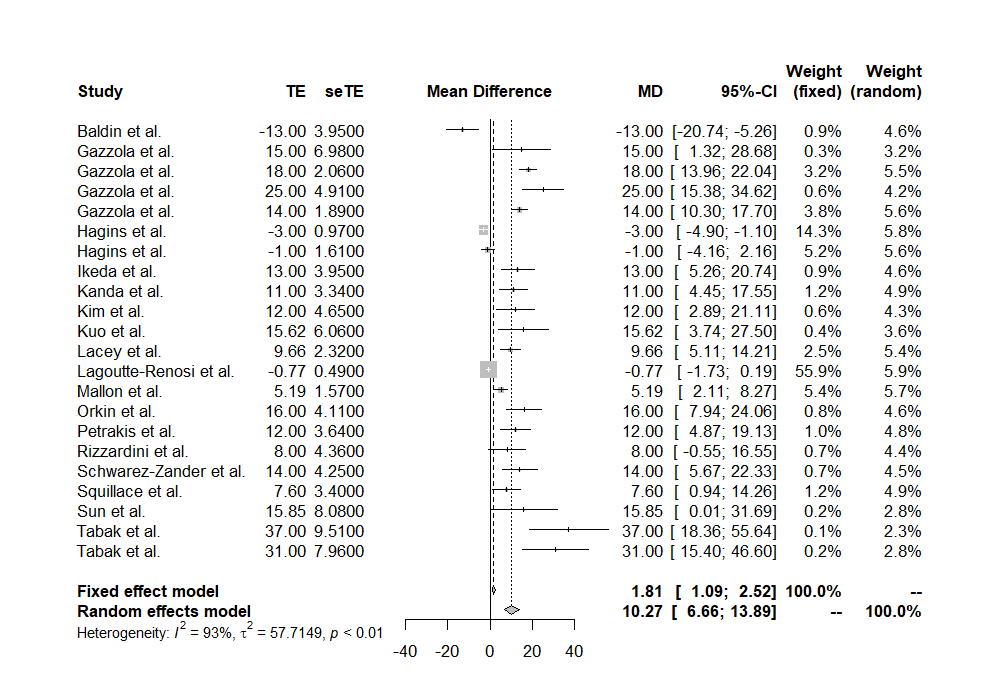 | 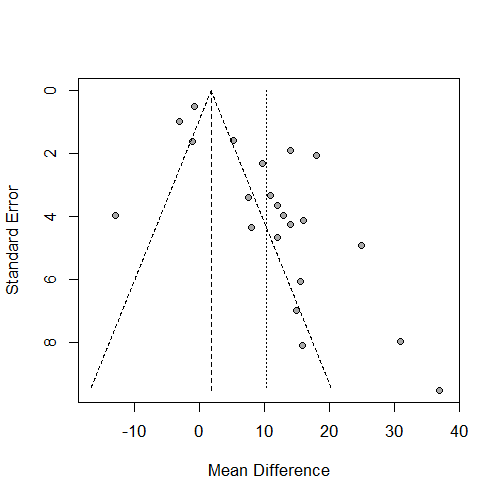 |
| LDL  12-mo | 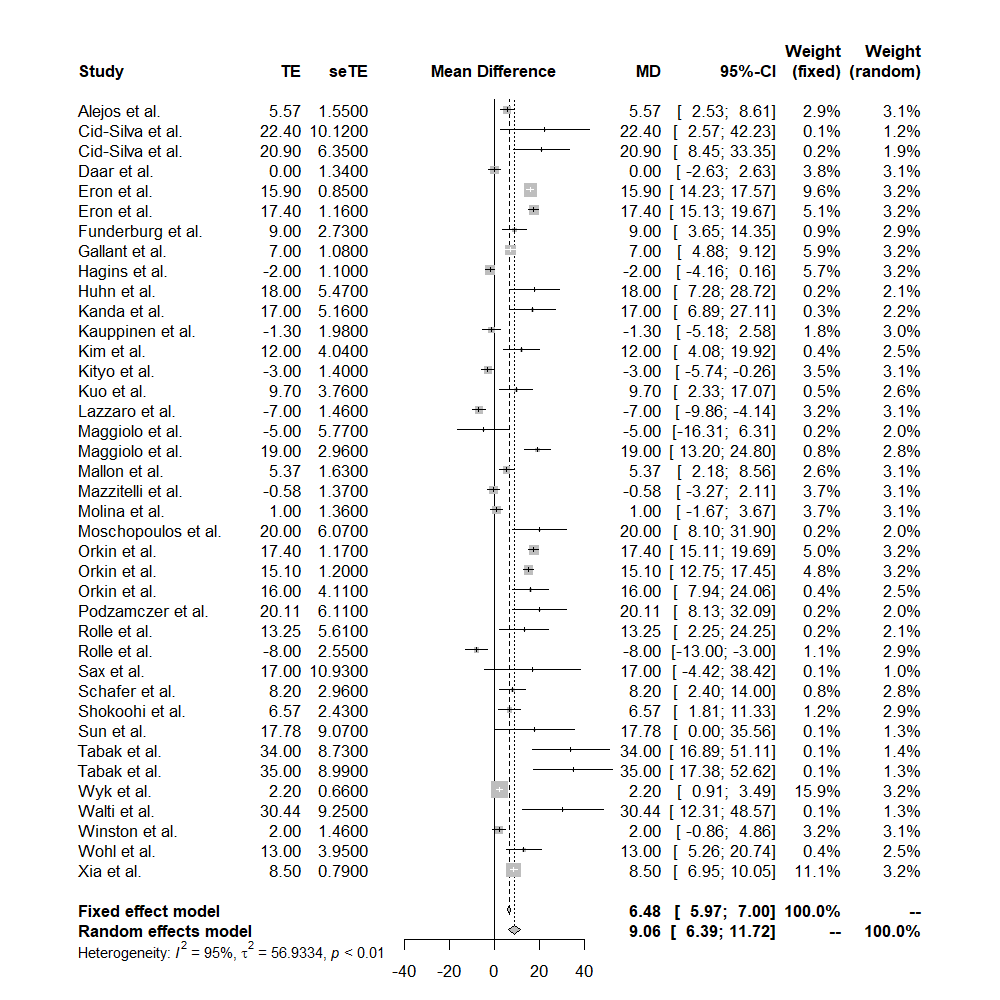 | 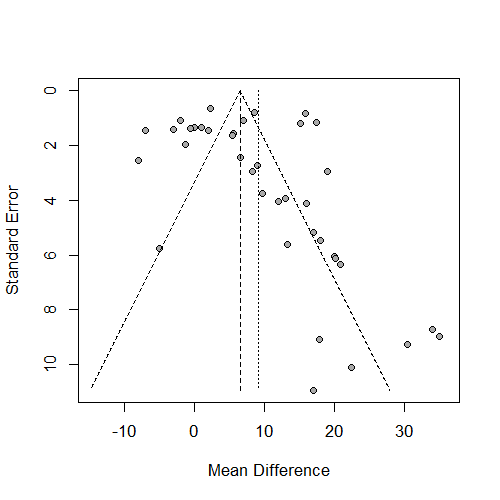 |
| LDL  24-mo | 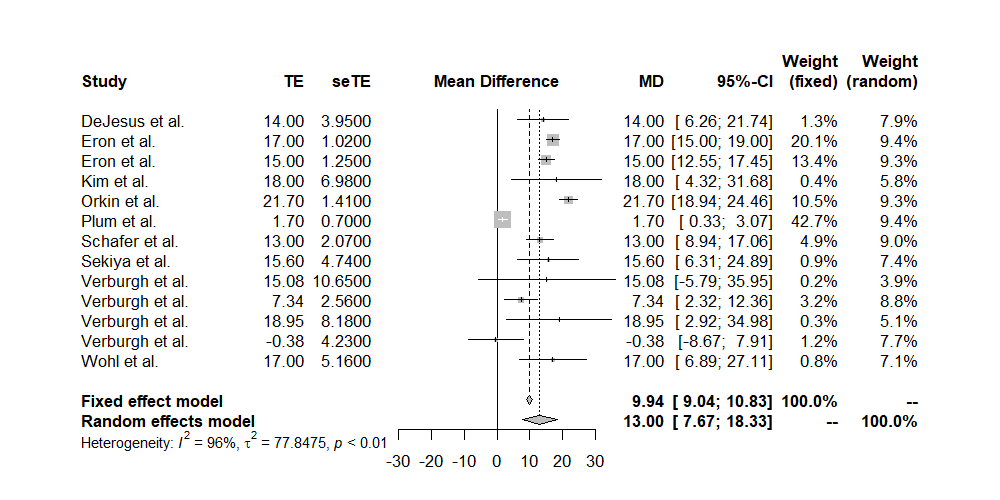 | 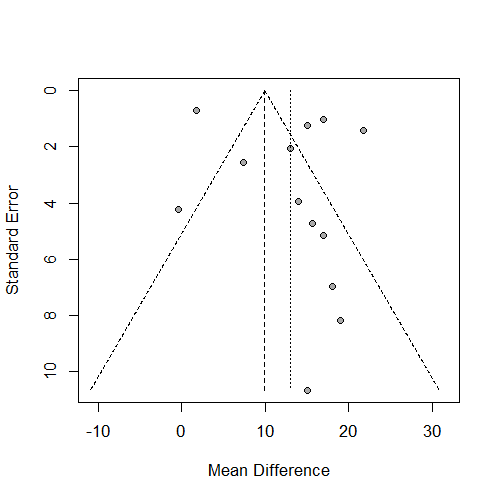 |
| LDL  36-mo | 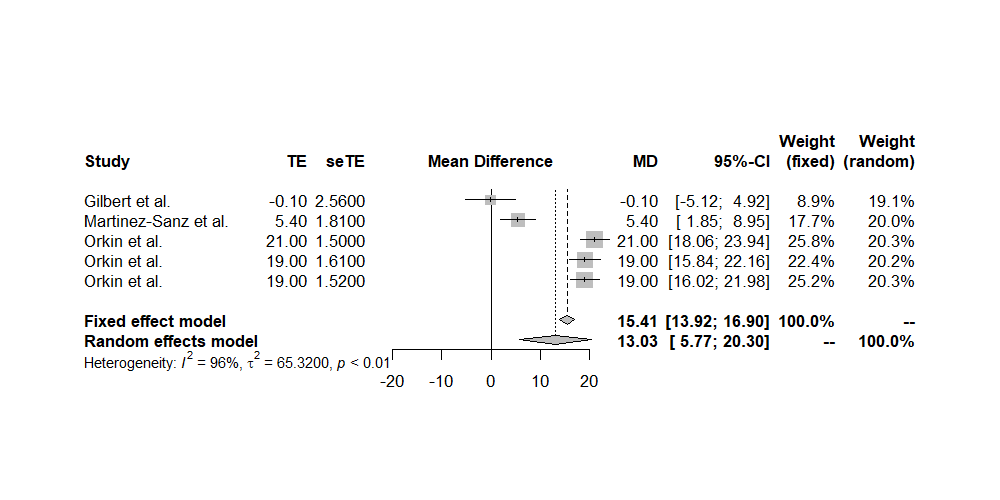 | 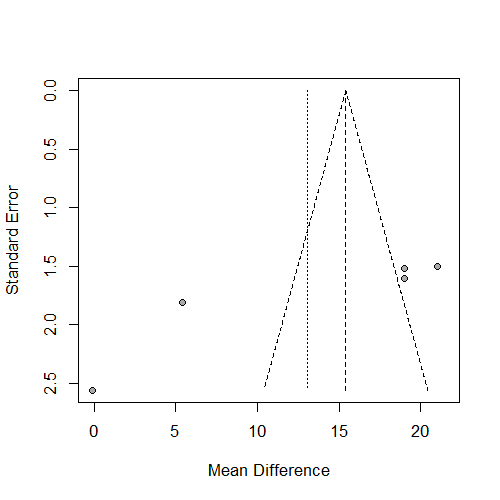 |
| Total cholesterol  3-mo | 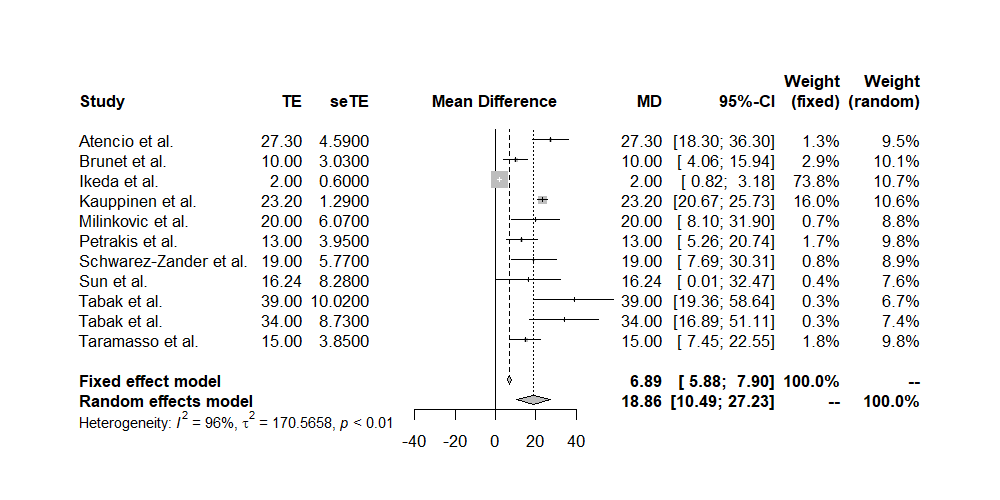 | 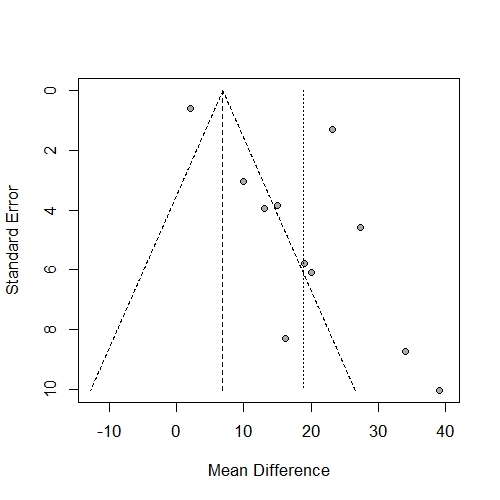 |
| Total cholesterol  6-mo | 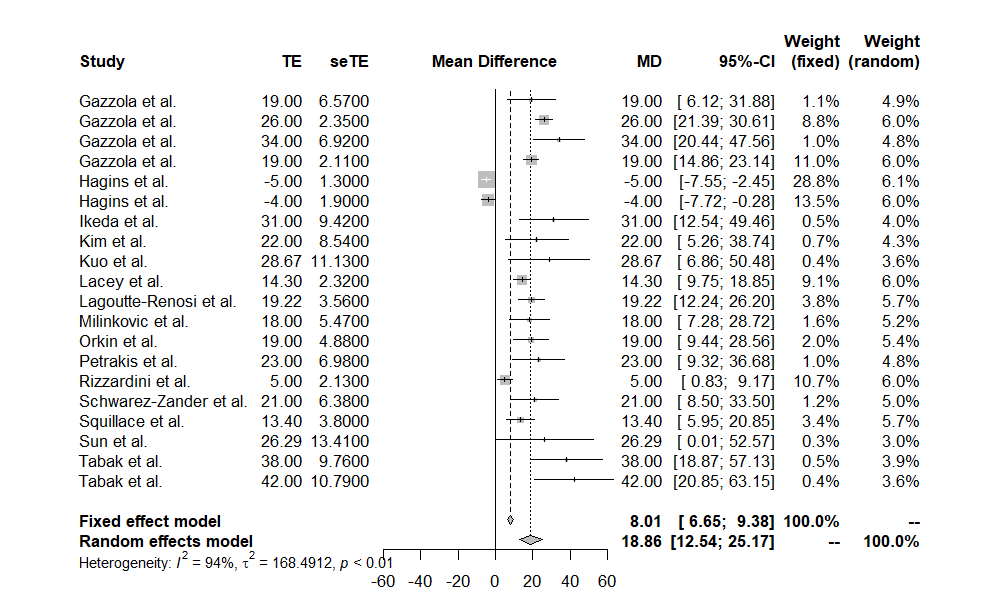 | 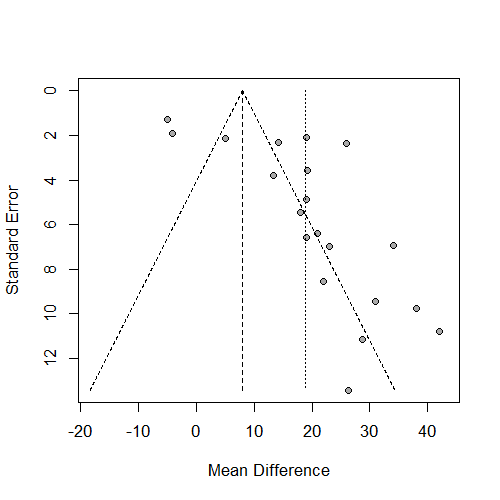 |
| Total cholesterol  12-mo | 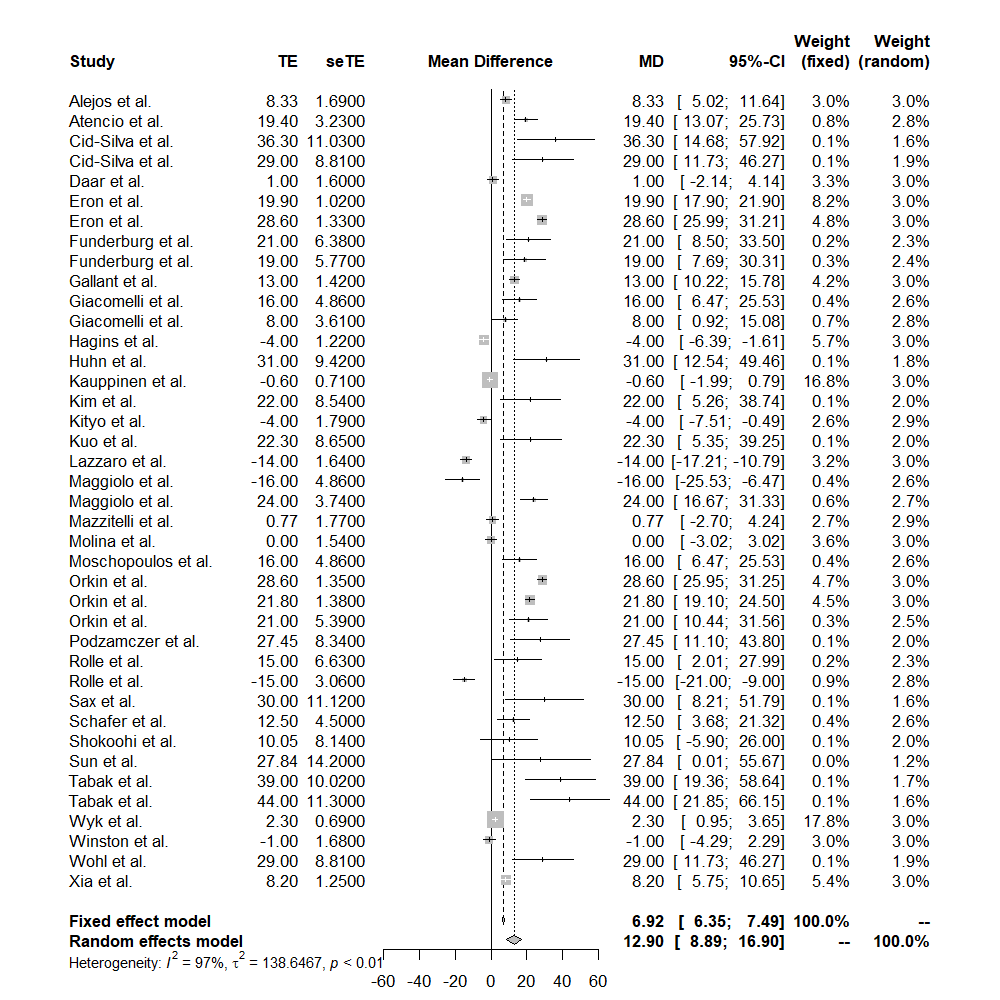 | 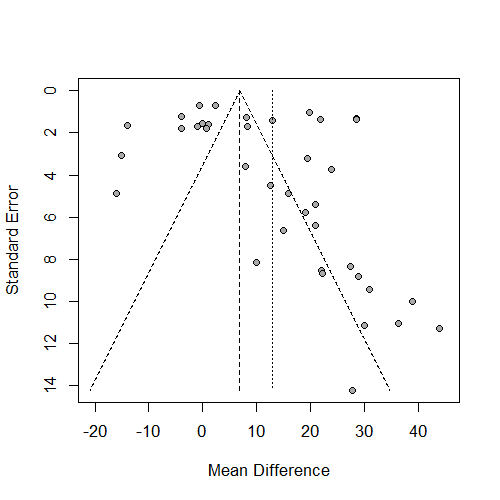 |
| Total cholesterol  24-mo | 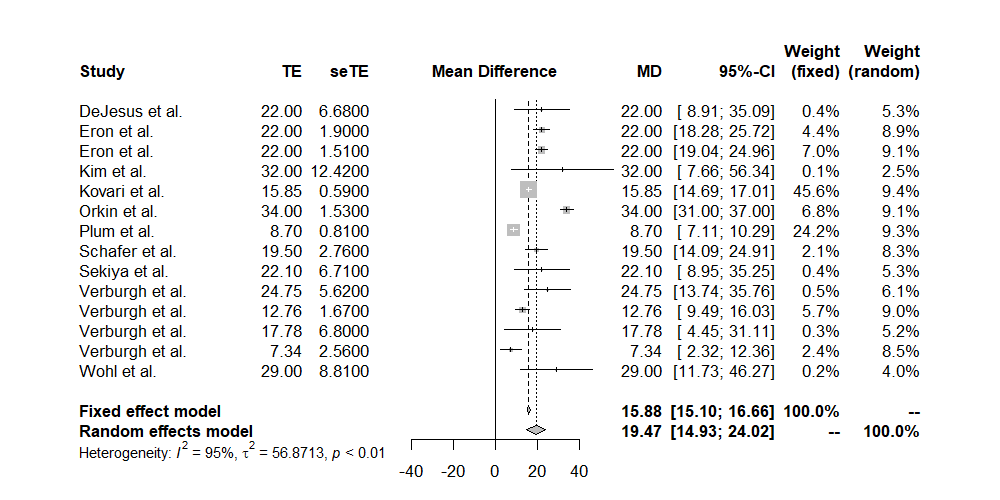 | 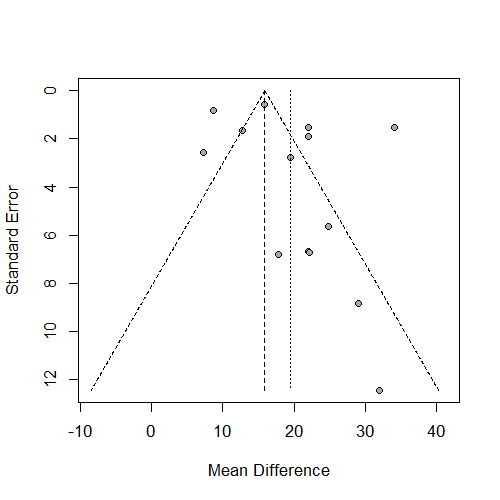 |
| Total cholesterol  36-mo | 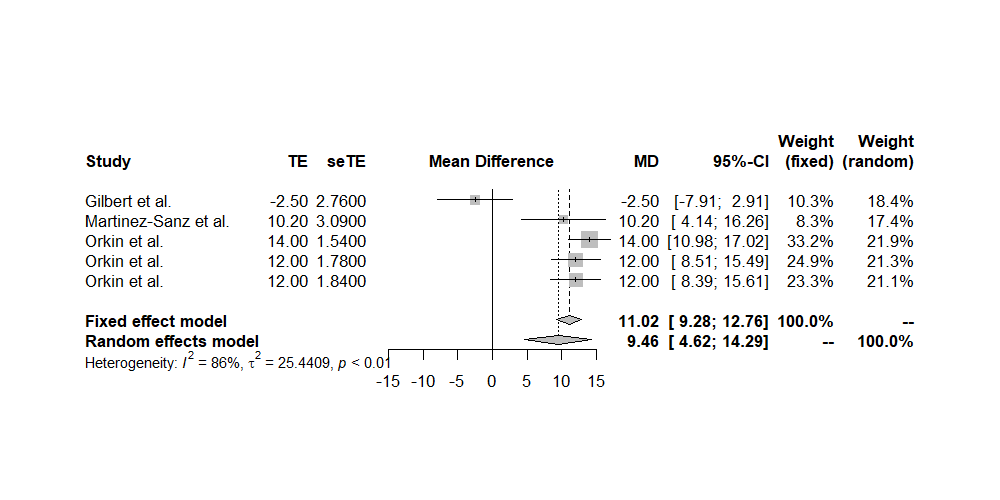 | 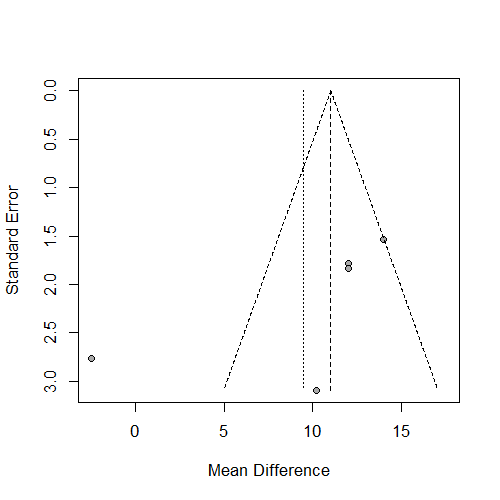 |
| Triglyceride  3-mo | 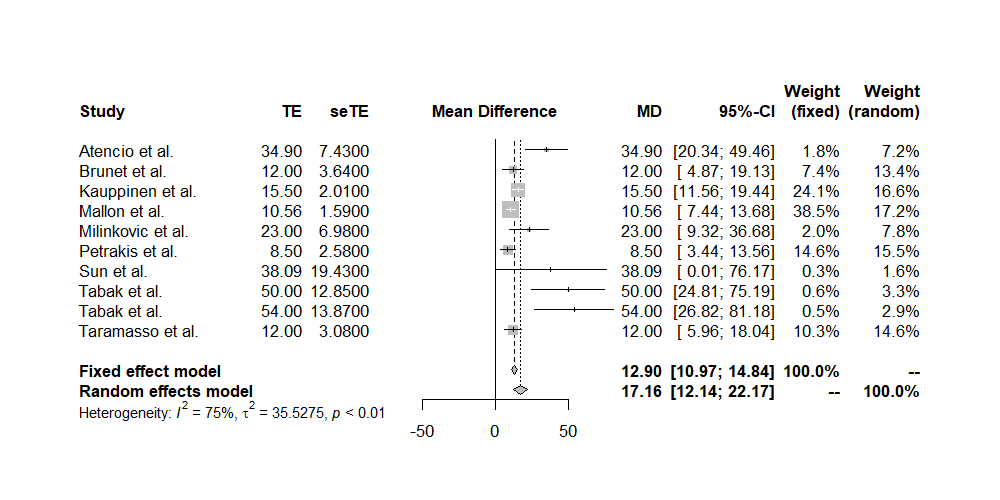 | 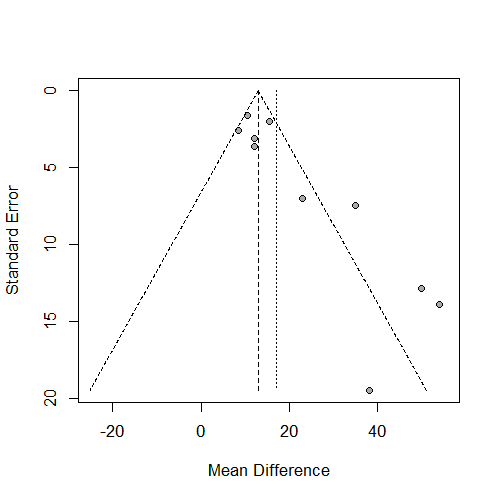 |
| Triglyceride  6-mo | 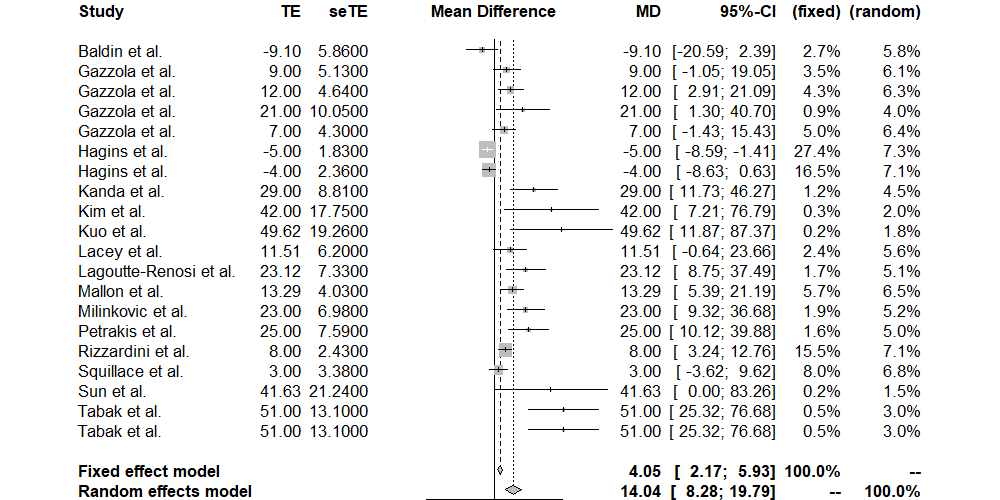 | 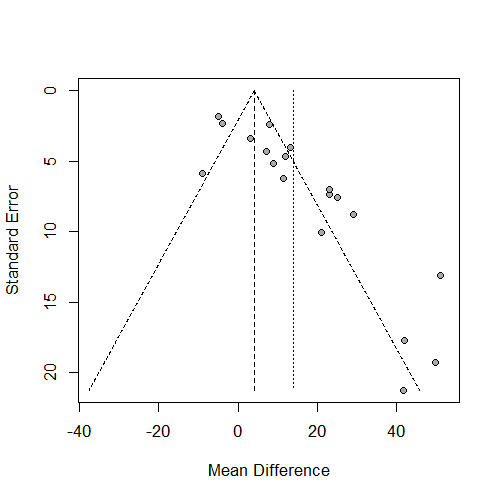 |
| Triglyceride  12-mo | 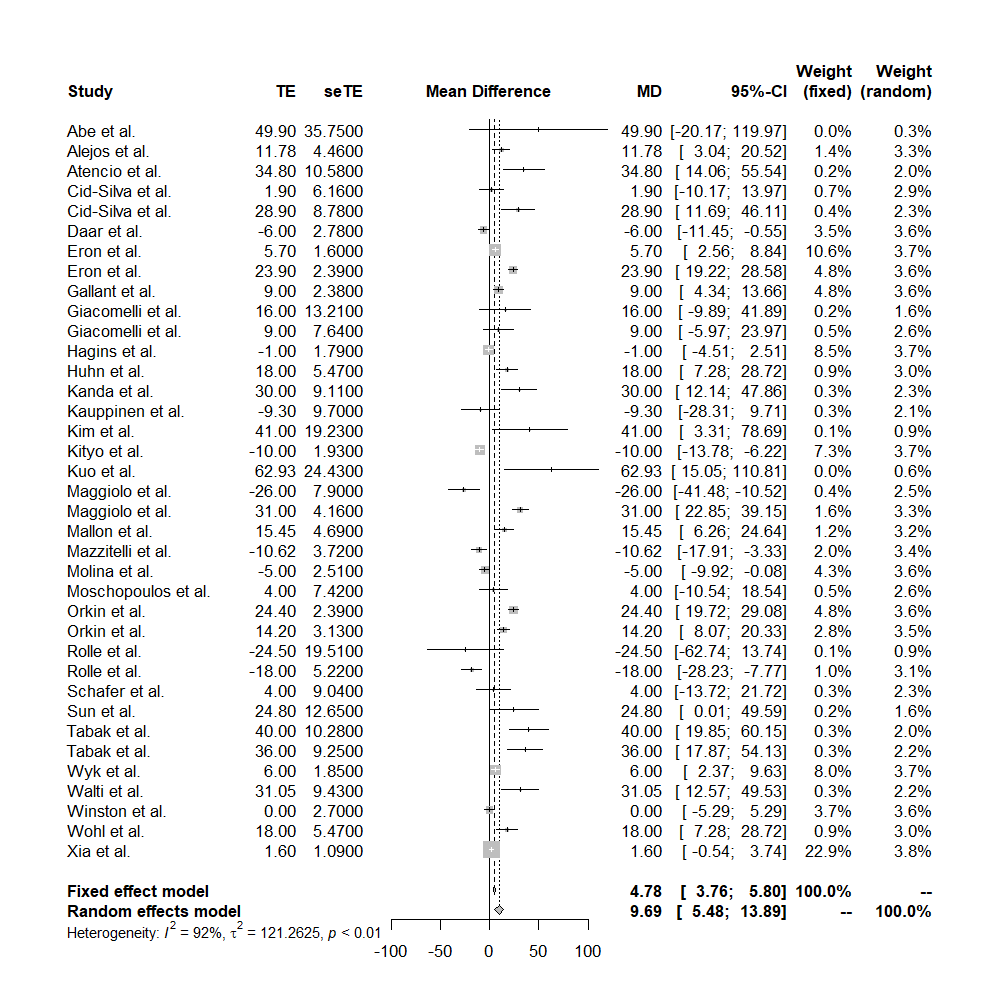 | 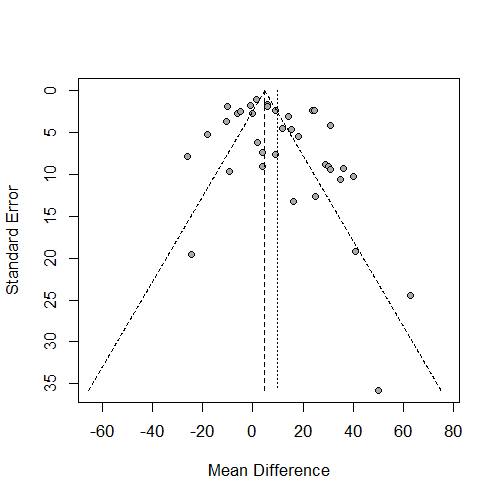 |
| Triglyceride  24-mo | 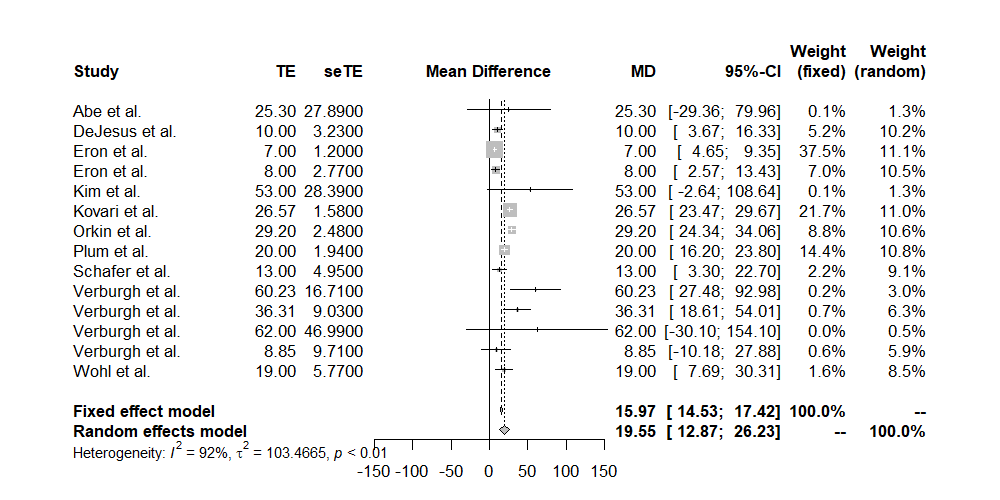 | 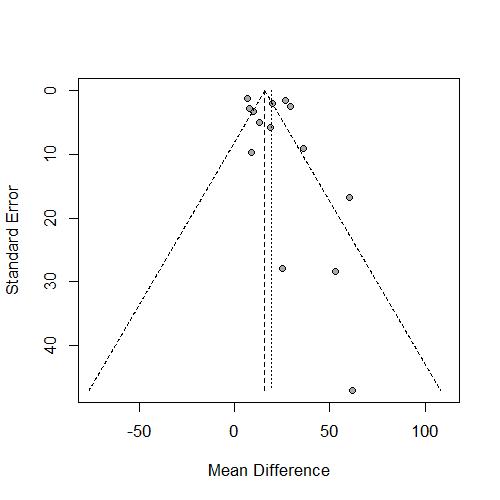 |
| Triglyceride  36-mo | 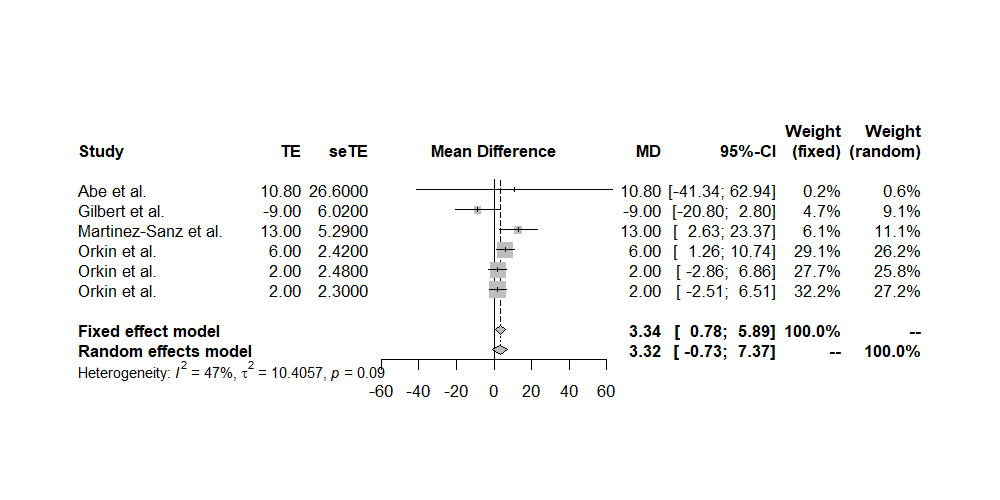 | 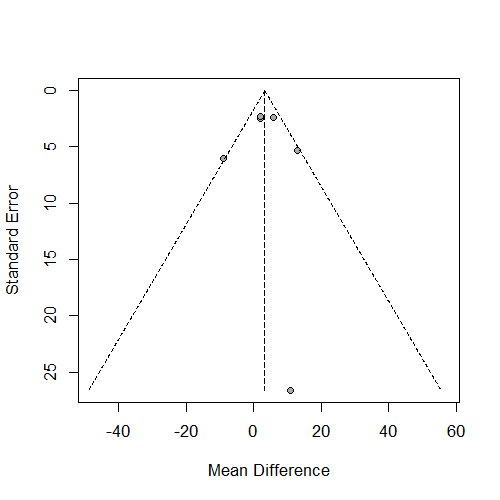 |

**Supplementary Figure 2. Forest plots and funnel plots of Table 5**

|  | Forest plots | Funnel Plots |
| --- | --- | --- |
| HDL  12-mo | 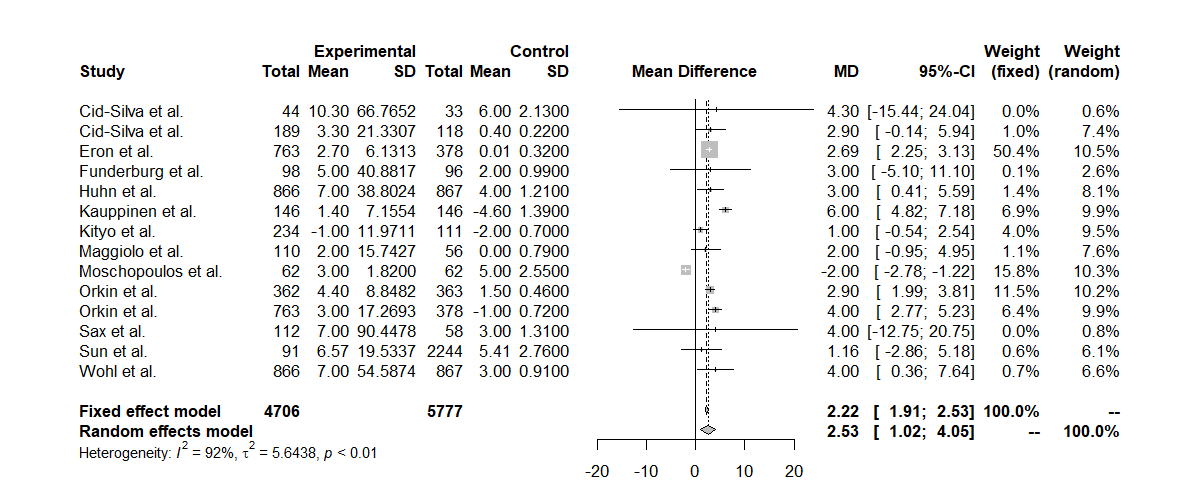 | 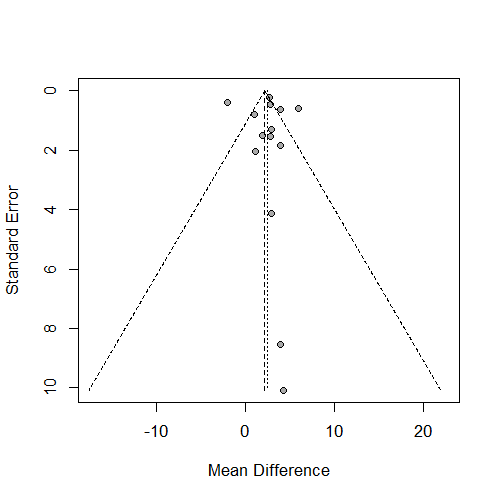 |
| LDL  12-mo | 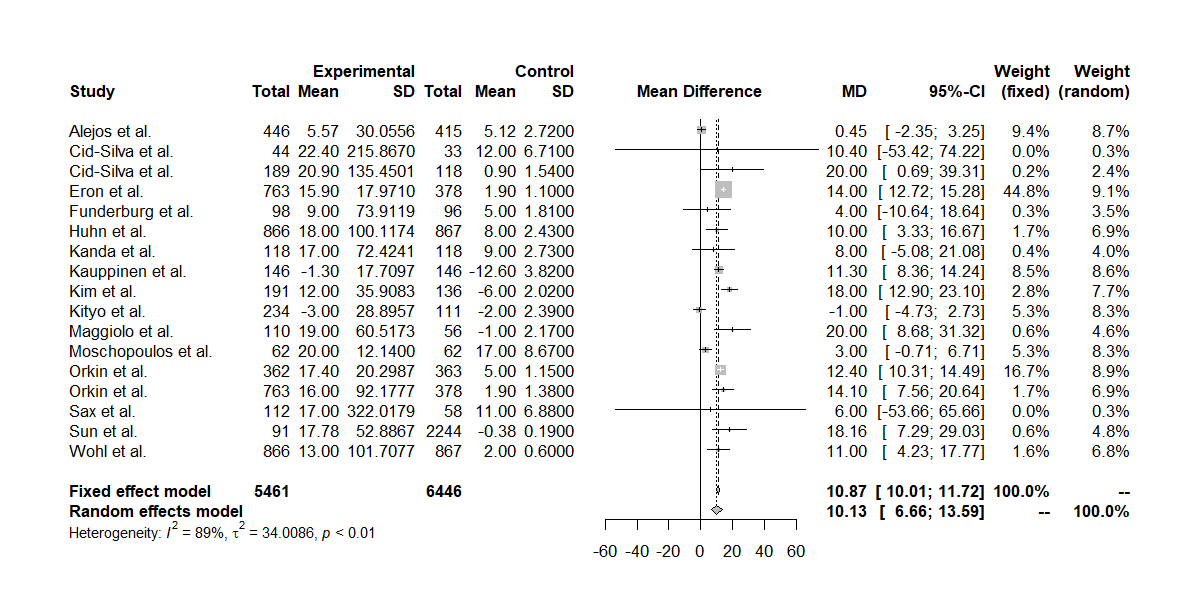 | 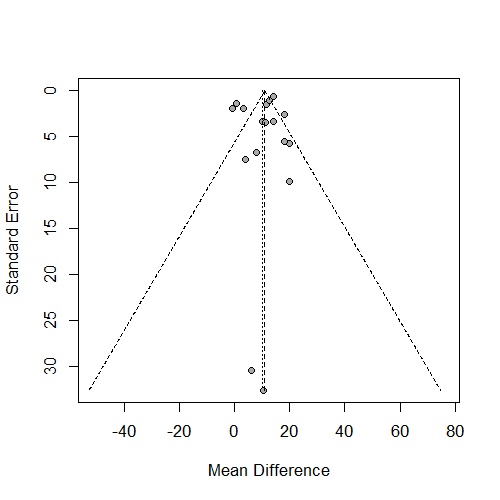 |
| Total cholesterol  12-mo | 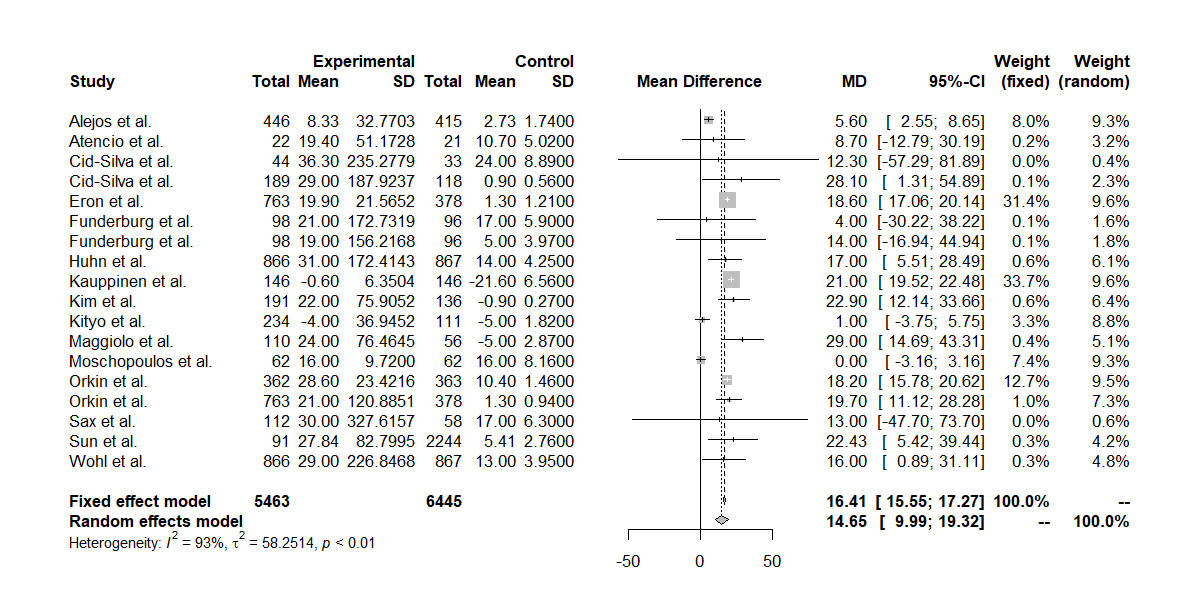 | 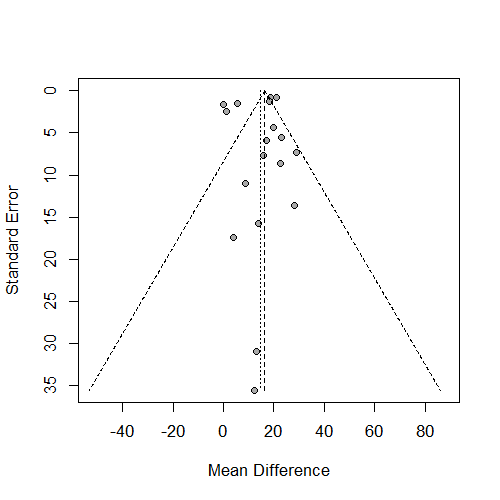 |
| Triglyceride  12-mo | 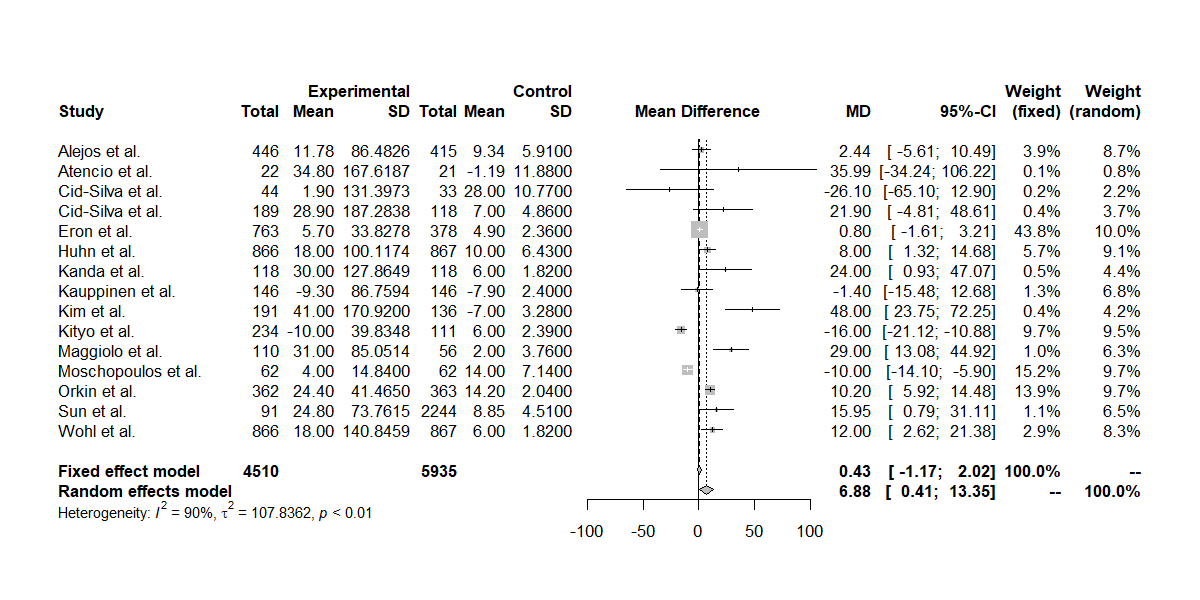 | 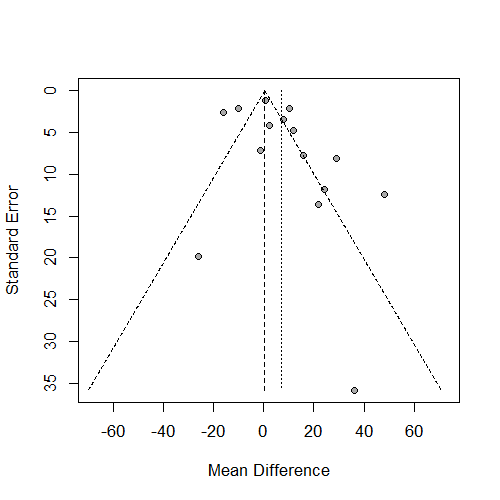 |

**Supplementary Figure 3. Forest plots and funnel plots of Table 6**

|  | Forest plots | Funnel Plots |
| --- | --- | --- |
| HDL  12-mo | 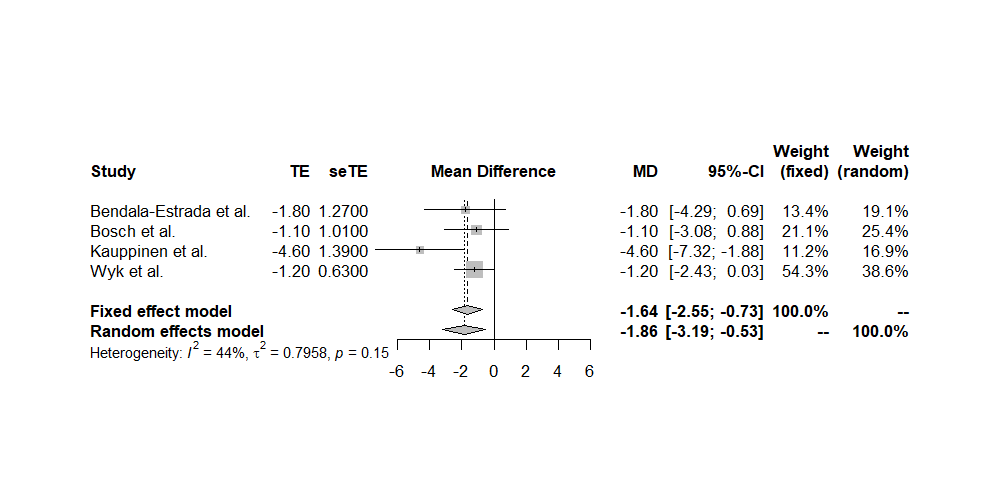 | 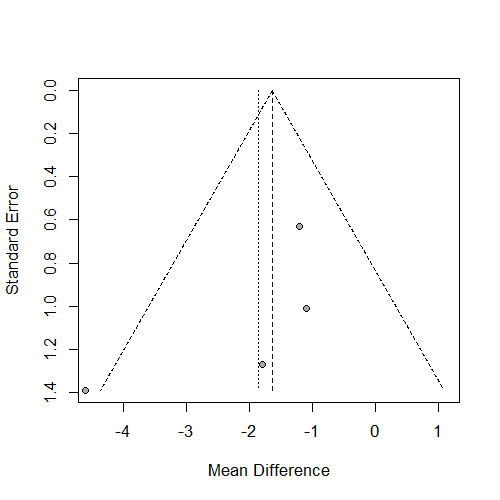 |
| LDL  12-mo | 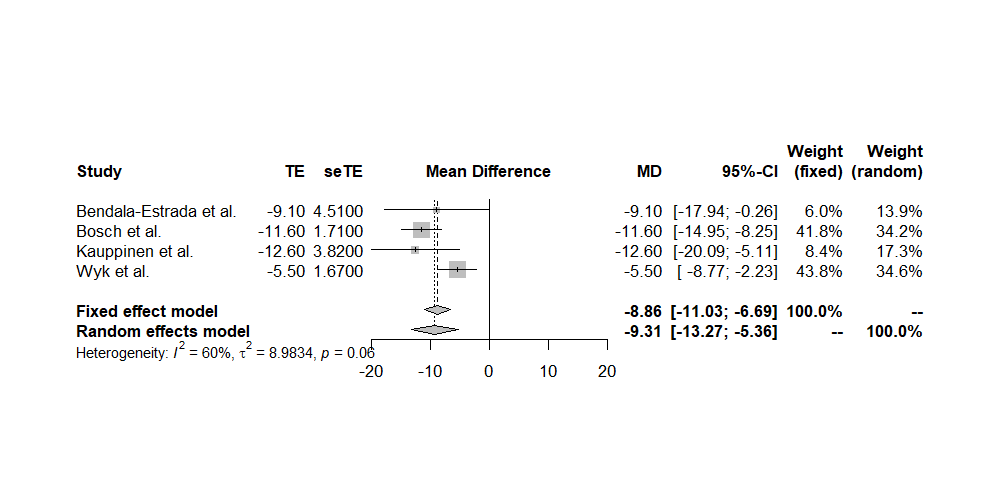 | 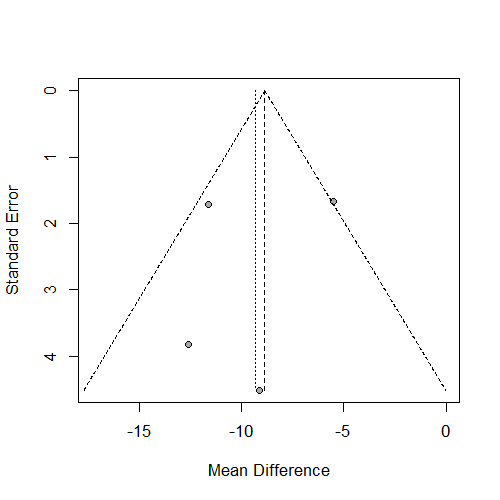 |
| Total cholesterol  12-mo | 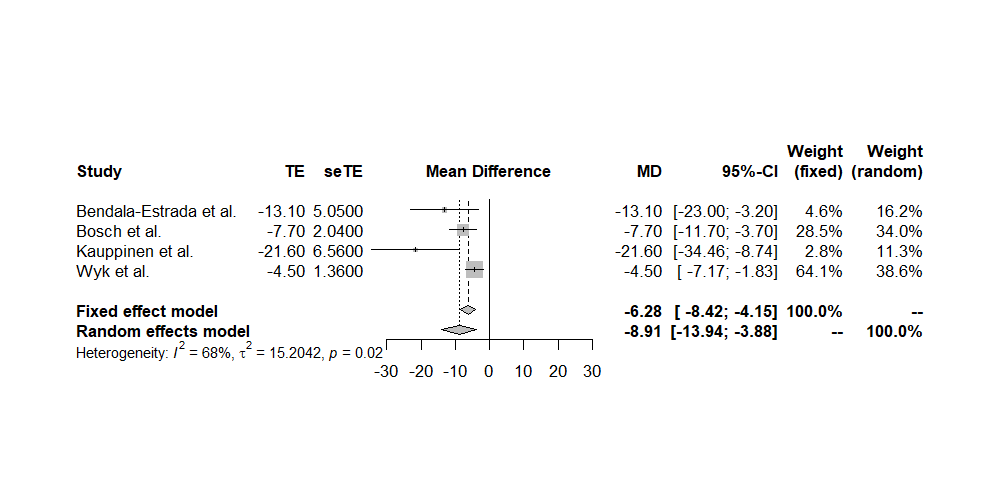 | 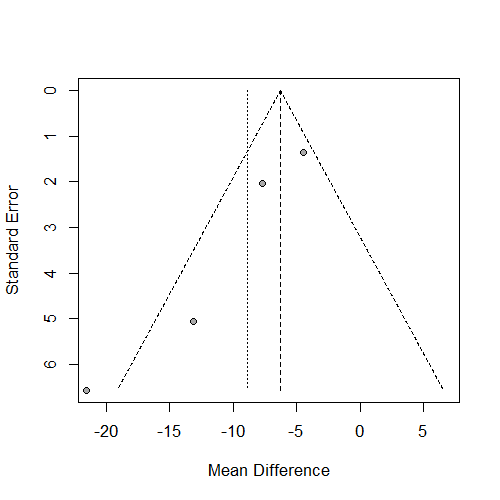 |
| Triglyceride  12-mo | 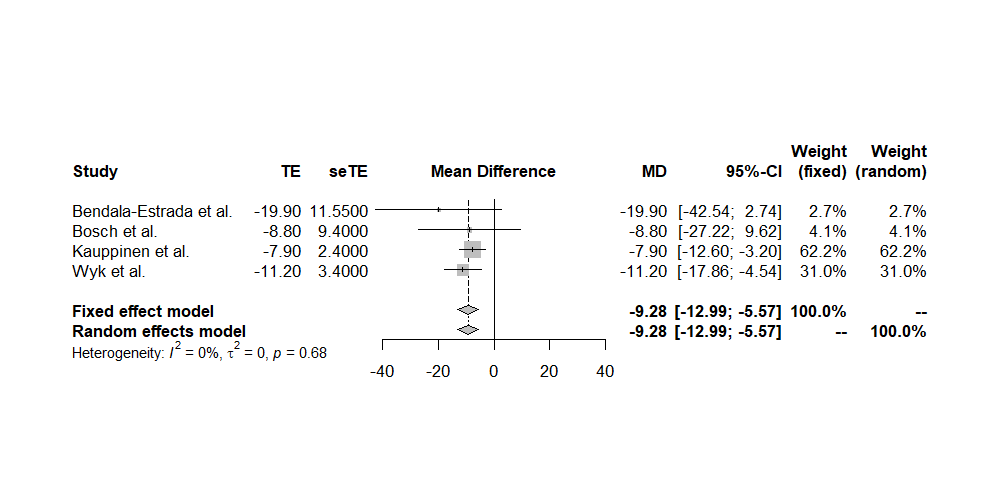 | 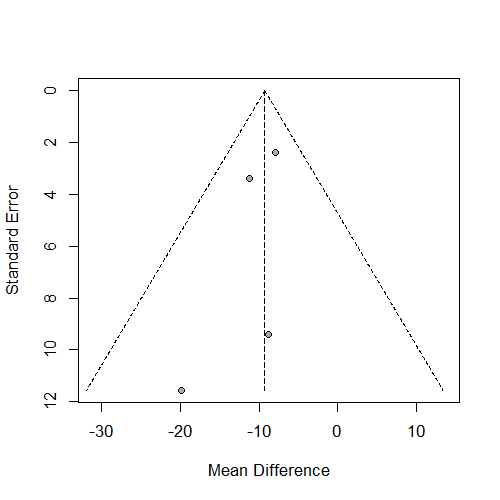 |

**Supplementary Figure 4. Forest plots and funnel plots of Table 7**

|  | Forest plots | Funnel Plots |
| --- | --- | --- |
| Overall period | 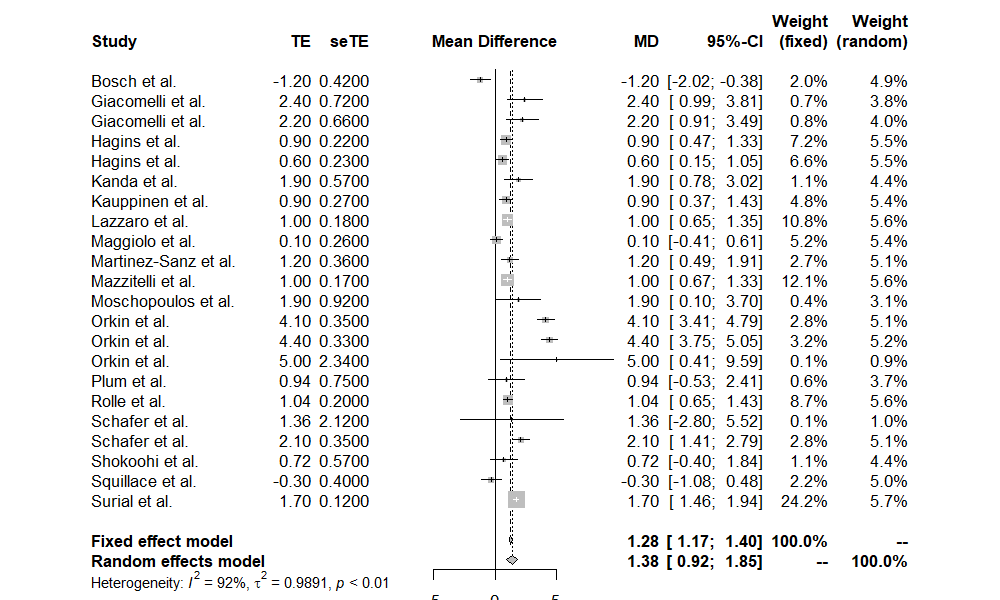 | 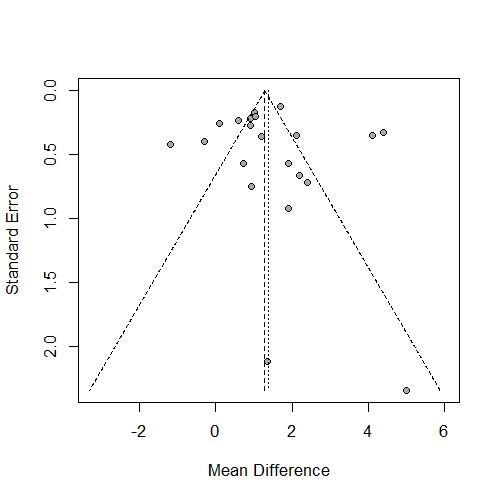 |
| 6-mo | 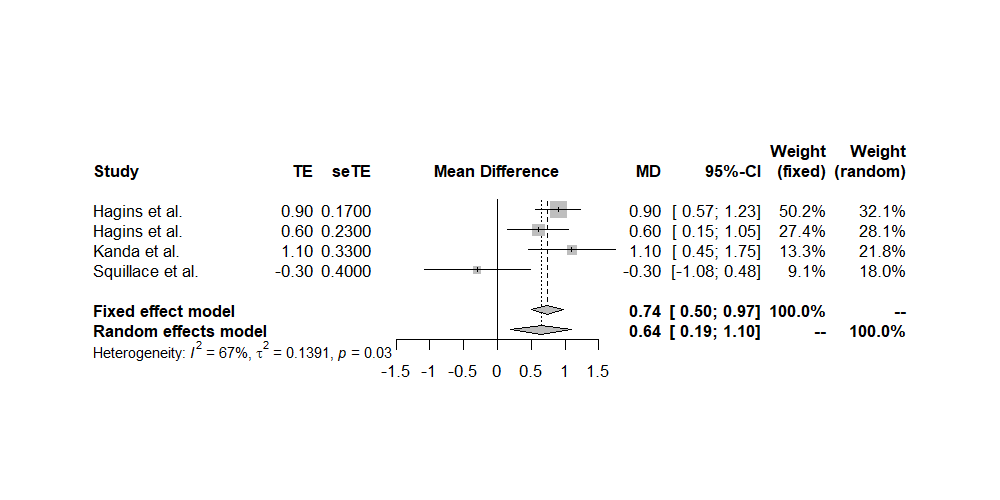 | 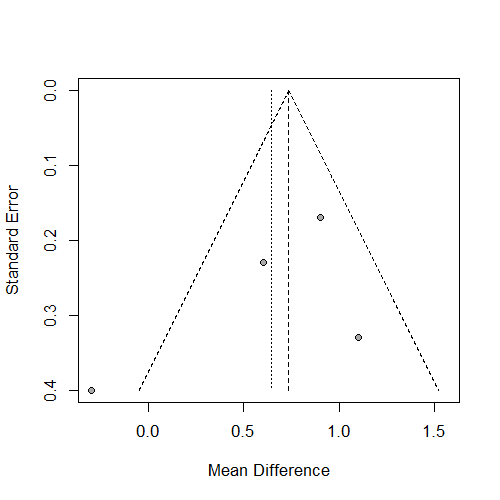 |
| 12-mo | 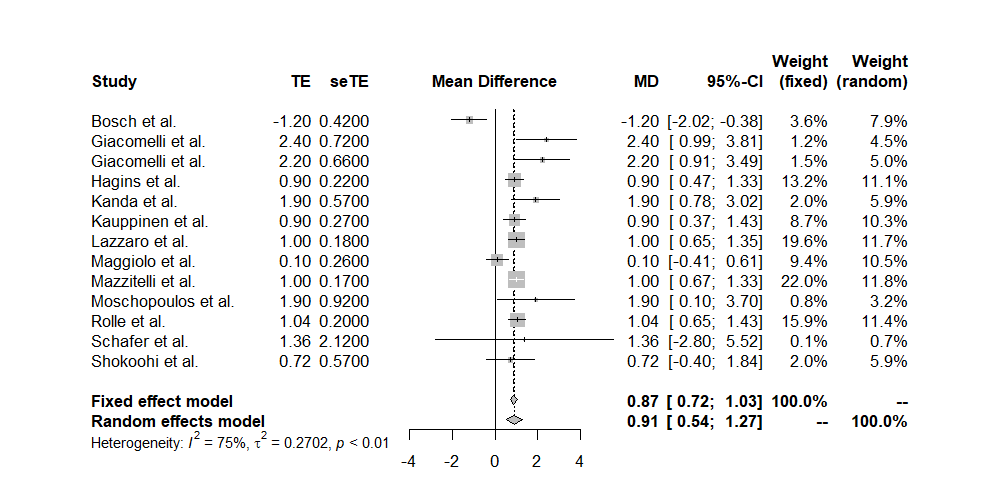 | 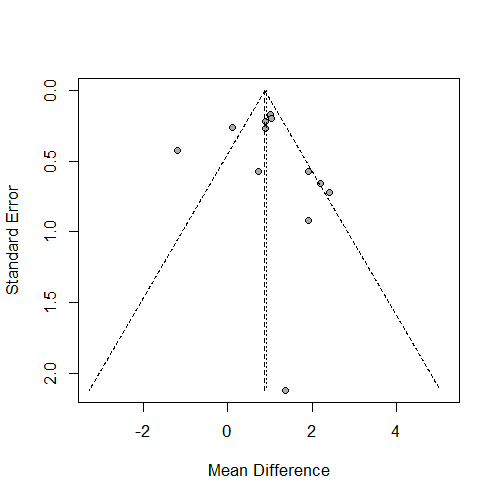 |
| 24-mo | 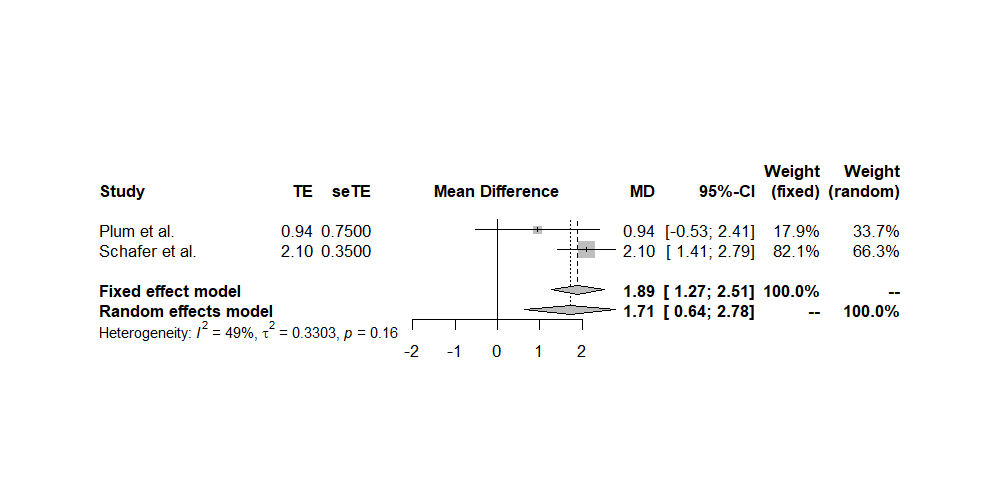 | 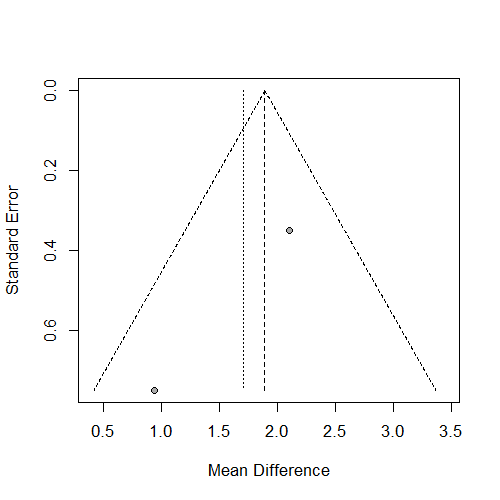 |
| 36-mo | 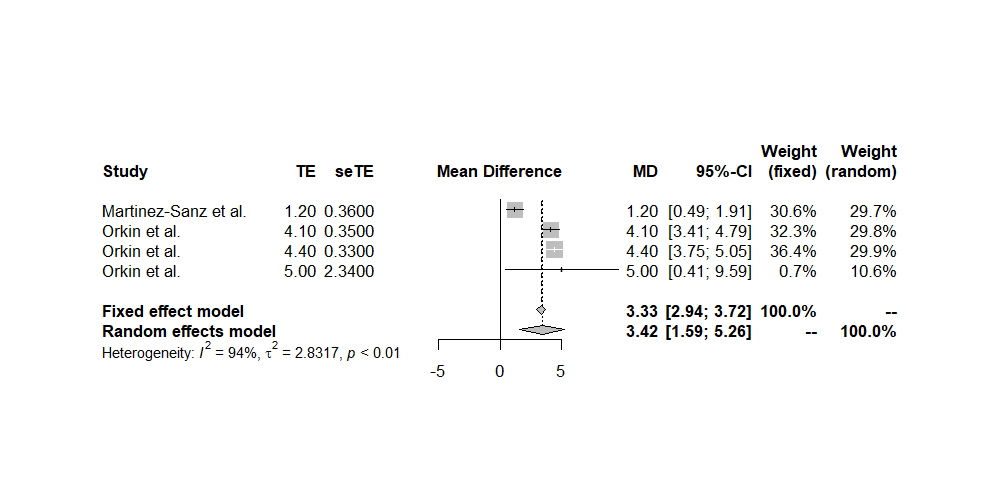 | 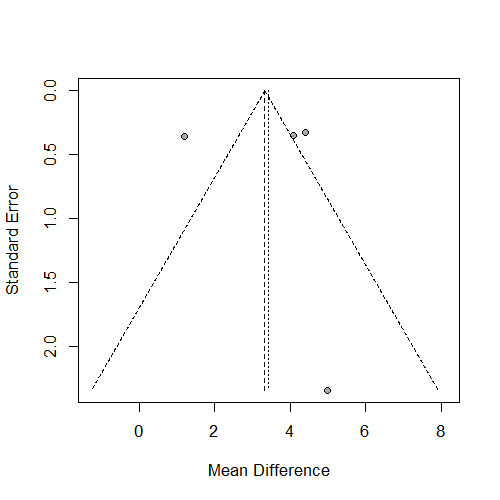 |
